# Arid Soil Bacterial Legacies Improve Drought Resilience of the Keystone Grass, *Themeda triandra*

**DOI:** 10.1111/mec.70062

**Published:** 2025-08-18

**Authors:** Riley J. Hodgson, Christian Cando‐Dumancela, Tarryn Davies, Victoria Drysdale, Nicole W. Fickling, Craig Liddicoat, Shawn D. Peddle, Sunita A. Ramesh, Declan Spoor, Alex Taylor, Carl Watson, Martin F. Breed

**Affiliations:** ^1^ College of Science and Engineering Flinders University Bedford Park South Australia Australia; ^2^ Adelaide South Australia Australia

**Keywords:** amplicon sequencing, drought resilience, endosphere, rhizosphere, soil microbiota, two‐step selection

## Abstract

Plant–microbe interactions are critical to ecosystem functioning and impact soil legacies, where plants exert a lasting influence on the microbial and physicochemical conditions of the soils in which they grow. These soil legacies can affect subsequent plant growth and fitness. Specifically, biotic soil legacies can influence microbially associated plant fitness through the movement of soil microbiota in a two‐step selection process: Microbes are recruited from bulk soil into the rhizosphere (the space around roots) and then into the endosphere (within plant roots). Furthermore, these endosphere root microbiota can also influence plant behaviour, shaping bulk soil communities over time. However, the potential of these soil legacies to provide host plant drought tolerance remains poorly understood. In a drought stress greenhouse trial, we show that arid soil legacies increased the biomass of the keystone grass *Themeda triandra* under both drought and control conditions. We report strong positive associations between *T. triandra* biomass and bacterial alpha diversity across soils, rhizospheres and endospheres. These findings show that bacterial soil legacies have an important but underappreciated role in grassland species resilience to drought and could be better harnessed to support resilient grassland restoration efforts.

## 1 Introduction

Grasslands are in significant global decline (Bardgett et al. 2021), and new solutions are required to restore the productivity, diversity and resilience of these ecosystems. Soil microbiota are key organisms involved in each of these components and are also involved in many resultant grassland ecosystem services (Koziol and Bever 2017; Wang et al. 2019; Liu et al. 2022). Despite strong plant–soil interactions in grasslands (i.e., plant–soil feedbacks), these interactions are underutilised in conservation and restoration efforts (Robinson et al. 2023; Peddle et al. 2024). As climate change and land‐use pressures intensify, understanding how soil microbiota support grassland productivity and stress tolerance is increasingly important to aid these conservation and restoration efforts (Trivedi et al. 2022; Fadiji et al. 2023).

Carbon and nutrient cycling are among the many microbial‐driven processes in soil that can shape plant communities (Bever et al. 2010; Wagg et al. 2014), especially in grasslands (Liao et al. 2023). In addition to these high‐level influences, plants also form direct symbioses with soil microbiota in their rhizospheres (areas around plant roots) and endospheres (inside plant roots) (Bulgarelli et al. 2013). Soil bacteria in grasslands have been shown to respond to different grassland management strategies at large scales, and microbial heterogeneity can impact vegetation dynamics (Sayer et al. 2013). However, grassland microbial communities vary by soil depth (Potthoff et al. 2006), grass species and soil types (Singh et al. 2007). The colonisation of rhizosphere and endosphere compartments by soil microbiota is described by the two‐step selection process (Bulgarelli et al. 2012, 2013; Lundberg et al. 2012). In the first step, the initial provision of resources via root exudates can support microbial assemblages from the bulk soil to establish in the rhizospheres. Secondly, microbiota are filtered into the endosphere with plant immune system regulation (Bulgarelli et al. 2013). These rhizosphere and endosphere microbiota aid in nutrient acquisition and plant metabolism, but we currently lack a clear understanding of how recruitment is affected by plants growing under stressful conditions, such as drought. We also lack knowledge of how plant recruitment of these microbiota is affected by ecological contexts (e.g., high vs. low aridity) (Ling et al. 2022; Santoyo 2022).

Harnessing soil biodiversity is increasingly recognised for its potential to enhance plant growth in applied ecology contexts (Mariotte et al. 2018; Porter and Sachs 2020; Peddle et al. 2024). One promising method to do this is through whole soil inoculations via the translocation of soil, including its microbiota, into new areas (Gebhardt et al. 2017; Wolfsdorf et al. 2021; Han et al. 2022). This approach leverages positive soil legacies where plant populations naturally cultivate soil microbiota that support the offspring of these plants (Kaisermann et al. 2017; Pineda et al. 2017; Buchenau et al. 2022). Positive soil legacies can improve plant tolerance to water stress and herbivory via the microbiota (Kaisermann et al. 2017; Hannula et al. 2021), but we lack a theoretical understanding of microbial colonisation mechanisms between soil and plant compartments. Experimental testing of how different soils, their microbiota and endosymbiotic root bacteria influence plant growth, combined with comprehensive characterisation of bacterial colonisation patterns, can help address these knowledge gaps, particularly under stress scenarios.

*Themeda triandra* (Forssk.) is a globally important keystone C4 grass species with a pan‐palaeotropical distribution (Snyman et al. 2013; Dunning et al. 2017; Pascoe 2018). Despite its ecological importance, the processes by which microbiota colonise and influence *T. triandra* remain poorly understood. Research to date has shown that *T. triandra* responds positively to regular disturbance, such as periodic fire, grazing or mowing (Morgan and Lunt 1999; Price et al. 2019), but is sensitive to overgrazing and extreme disturbance (Snyman et al. 2013). It is also known for its drought tolerance (Snyman et al. 1997). While soil fungi have been shown to enhance its growth under drought conditions (Petipas et al. 2017), the contributions of soil bacteria remain unexplored, especially in arid areas (Hodgson et al. 2025). To address this, we conducted a greenhouse experiment on how soil microbiota from high and low‐aridity regions affected the germination and growth of *T. triandra* under both water‐available and drought‐like (i.e., water stress) conditions.

In this study we used 16S rRNA amplicon sequencing to characterise the *T. triandra* ‐associated microbiota of high and low‐aridity soils under live versus sterilised, and water stress treatment conditions, plus the recruitment patterns of these microbiota from the bulk soils into *T. triandra* rhizospheres and endospheres. We hypothesised that: (1) soil microbiota sourced from arid sites would enhance *T. triandra* growth under stress conditions by providing mutualistic microbiota that support growth under drought‐like conditions; (2) distinct microbial communities would be recruited into the rhizosphere and endosphere under each water treatment, reflecting shifts in host plant requirements; and (3) the presence of *T. triandra* plants would alter the bacterial community in soil due to a cumulative influence of microbe–root interactions. By assessing how microbiota impact the drought responses of this important grass, and monitoring their recruitment across root compartments, we can better understand the value of soil biodiversity as a tool for improving the resilience of grassland ecosystems.

## 2 Materials and Methods

### 2.1 Experimental Design

We prepared a germination and 55‐month growth trial to test the influence of microbiota in soils collected from either high‐aridity or low‐aridity locations by growing *T. triandra* under sterilised and live microbiota conditions. We also assessed the germination and growth of *T. triandra* plants in these soils under water‐available versus water stress conditions (mimicking a drought). Each of the eight treatments (i.e., 2 × soil aridity levels, 2 × sterilised, 2 × water availability) had 10 replicate pots, making 80 pots in total (see Figure S1a,b). Each pot received an equal 1190 g dry weight of its assigned soil. We calculated the relative soil water content for each soil treatment to provide a standardised measure of moisture, with 0% corresponding to oven‐dry soil and 100% to maximum water‐holding capacity. For the control treatment, we watered each pot to 100% relative soil water content, while we maintained the water stress treatment at 40% by regularly weighing and watering, following the method described by Earl (2003). Relative soil water content was calculated by weighing whole pots (subtracting pot weight) and comparing the mass of moist soil at the time of measurement to the mass of oven‐dried soil (dried at 105°C for 72 h) and the mass at field capacity, which was determined at the start of the experiment. Values were expressed as percentages of field capacity.

Relative soil water content generally ranged from 75%–88% in the control (no‐stress) treatment and from 35%–38% in the water stress treatment (Figure S2). We also included an additional 24 soil‐only pots to account for changes in microbiota across each treatment in the absence of *T. triandra* (Figure S1c).

**FIGURE 1:**
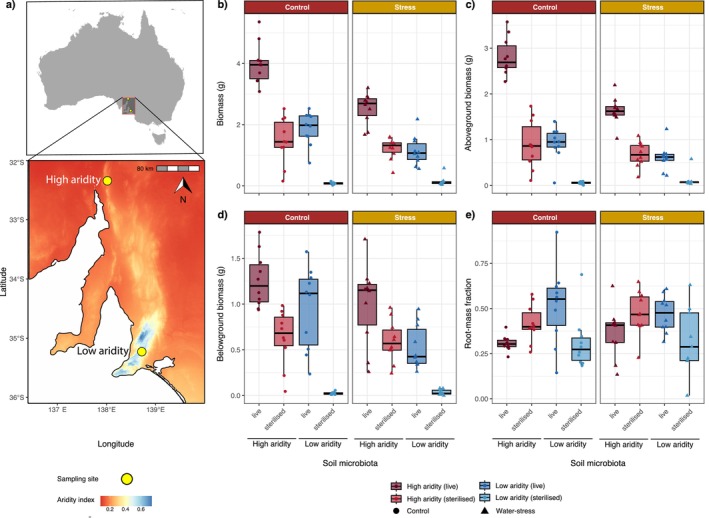
High‐ and low‐aridity sampling sites, and *Themeda triandra* plant trait responses to treatment effects. (a) High‐ and low‐aridity sampling sites for the collection of soil microbiota for experimental manipulation (yellow points). Mean annual aridity index data layer (ADM) is sourced from the Soil and Landscape Grid of Australia (Searle et al. 2022), where aridity index is calculated via annual precipitation/annual potential evaporation. *T. triandra* plant growth responses to soil aridity, sterilised and water stress treatments showing: *T. triandra* (b) total biomass, (c) aboveground biomass, (d) belowground biomass and (e) root–mass fraction differences. We chose not to overlay statistical groupings on raw‐data boxplots, as differences were tested via a mixed model that accounts for random structure (e.g., soil aridity). Statistical comparisons and groupings based on model‐estimated differences are provided in Table S6.

To capture naturally occurring soil microbiota associated with *T. triandra* , bulk soil was collected from around the roots of *T. triandra* plants in two undisturbed remnant sites (Figure 1a) with different levels of aridity: Kuitpo Forest Reserve at 35.2279° S, 138.7199° E (the mesic, low‐aridity site; aridity index = 0.658—henceforth low‐aridity soil) and Quorn Floral Reserve at 32.3434° S, 138.0182° E (the semi‐arid, high‐aridity site; aridity index = 0.227—hereafter high‐aridity soil) on 14 and 16 November 2023, respectively (Table S1). All seeds were collected from the remnant *T. triandra* in Kuitpo Forest Reserve in December 2020 (35.2279° S, 138.7199° E). After collection, soils were sieved at 5 mm to remove large stones and litter and then stored at 4°C for 1 month prior to setting up the growth trial. We avoided cross‐contamination by sterilising all equipment, including sieves, between contact with each soil, and followed laboratory field and laboratory protocols outlined in pre‐established methods (Cando‐Dumancela et al. 2021, 2023). Half of the soil volumes were sterilised by autoclaving them twice at 121°C, 2 days apart.

### 2.2 Germination and Growth Trial

The greenhouse was set at 16–8 h day‐night cycle, with temperatures set to 30°C and 18°C, respectively. In each pot (14 cm diameter, 2 L pots), we sowed 8 *T. triandra* seeds and recorded their weights before placing them in identifiable wells in each pot on 13 December 2023 (Figure S3; 80 seeds per treatment, 640 seeds total). We monitored seedling emergence rates across each treatment. After 8 weeks, seedlings were randomly thinned to one plant per pot (6 February 2024), and water stress conditions were imposed at 10 weeks (21 February 2024). At the conclusion of the experiment (23 weeks; 21 May 2024), we recorded aboveground and belowground biomass, root‐mass fraction (Pérez‐Harguindeguy et al. 2013) and relative interaction index values. Soils were collected immediately postharvest for both physicochemical and bacterial community profiling in 40 pots (5 pots per treatment). We also collected rhizosphere and endosphere samples from 40 pots for microbial profiling (described below).

### 2.3 Soil Physicochemical Analysis

We analysed the following soil physicochemical conditions from each sampling site before and from pots after the growth trial at CSBP Laboratories (Bibra Lake, Australia): phosphorus and potassium (Colwell 1965), sulphur (KCl 40) (Blair et al. 1991), organic carbon (Walkley and Armstrong 1934), nitrate and ammonium nitrogen (Rayment and Lyons method 7C2b), electrical conductivity and pH (CaCl_2_). The pH and electrical conductivity tests were performed by extracting soils in deionised water at 1:5 and stirring for an hour before measuring with an electrical conductivity electrode (Rayment and Lyons 2011). Calcium chloride was added to the equivalent of 0.1 M, and the pH CaCl2 was measured.

### 2.4 DNA Extraction, Sequencing and Bioinformatics

We prepared DNA extractions from *T. triandra* root endospheres following methods outlined in Hodgson et al. (2025). This involved cleaning the exterior of plant root surfaces by sonication at five 30 s on/off burst cycles in 0.02% Silwet L‐77 supplemented PBS buffer (pH = 6.5) for 5 min, followed by five 5 min washes in sterilised, distilled water. These methods underwent prior validation described in Hodgson et al. (2025). To extract microbial DNA from rhizospheres, we followed the protocol from McPherson et al. (2018). Briefly, root samples were washed in 0.02% Silwet L‐77 supplemented PBS buffer, vortexed for 45 min, and then filtered using 100 μm sieves (Thermo Fisher Scientific, Waltham, USA) and centrifuged at 1000 RPM. Soil samples taken from the plant plots at either the start or end of the trial were stored at −20°C after collection. DNA from soil, rhizosphere and endosphere samples was extracted using the DNeasy PowerLyzer PowerSoil Kit (Qiagen, Hilden, Germany), as per manufacturer's instructions.

Amplicon libraries of the 16S rRNA V3‐4 gene region were generated by the Australian Genome Research Facility (Brisbane, Australia) using the 341F (CCTAYGGGRBGCASCAG) and 806R (GGACTACNNGGGTATCTAAT) primers. Sequences were generated using the 300‐base pair paired‐end run of the Illumina NextSeq 2000 platform. The DADA2 bioinformatics pipeline was used to infer amplicon sequence variants (ASVs), with monotonicity enforced during error estimation. Additionally, loess smoothing was applied with specified weights, span and degree to improve error rate modelling. Qiime2 was used to identify profiles from amplicon sequence data from the SILVA database (v138.1) (Wang et al. 2007; Quast et al. 2013), using a naïve Bayesian classifier (Wang et al. 2007; Callahan et al. 2016; Bokulich et al. 2018). Taxa that were not assigned as Bacteria, unassigned at the phylum level and associated with mitochondria or chloroplasts were removed. We were unable to extract and sequence viable DNA concentrations from sterilised low‐aridity conditions at the beginning of the experiment, possibly due to the sterilising effects of autoclaving on microbiota and their DNA. Overall, we found a total of 25,687 unique ASVs with a median average of 44,740 reads per sample (13,357,259 reads total), which we reduced to 25,473 unique ASVs after rarefying to 18,738 reads per sample (3,485,268; see Table S4).

### 2.5 Statistical Analysis

All analyses were performed using R version 4.0.2 (R Core Team 2022).

#### 2.5.1 Soil Physicochemical Analyses

We used Kruskal–Wallis tests to assess differences in physicochemical soil conditions across soil types and experimental treatments. Pairwise comparisons were done using Dunn tests, with Holm adjusted *p* values at the 0.05 significance level.

#### 2.5.2 Germination and Plant Analysis

Seed germination across treatments was compared using generalised linear mixed‐effects models with a binomial link function with the R package lme4 (Bates et al. 2015). Soil aridity, sterilisation and seed mass were included as fixed effects, while the pot ID (8 seeds were sown per pot) was included as a random effect. To compare the differences in total biomass, aboveground biomass, belowground biomass and root‐mass fractions of *T. triandra* , we used randomised linear mixed‐effects models. Significance was determined by permuting each model 10,000 times and comparing observed test statistics with those of the simulated random distributions. We assessed model assumptions using diagnostic plots, including visual inspection of residual distributions and residual‐versus‐fitted plots, as well as the Shapiro–Wilk test for normality. While some models showed minor violations of normality or homoscedasticity, the nonparametric nature of our permutation‐based models allows for more robust statistical inference that is less sensitive to these assumptions. This provides greater confidence in the validity of our results where traditional parametric assumptions may not be fully met (Anderson 2001; Anderson and Robinson 2001).

For each plant response variable (i.e., total, aboveground and belowground biomass, and root‐mass fractions), we used linear mixed‐effects models to evaluate the effects of sterilisation and water stress treatments. In our initial models, sterilisation and water stress were treated as fixed effects, with soil aridity included as a random effect to account for variation among soils sourced across the aridity gradient. We tested both additive and interaction models to assess whether the effect of one treatment depended on the presence of the other. In a second set of models, we included soil aridity, sterilisation and water stress in additive and interaction terms, while treating sterilisation and water stress as random effects. This approach allowed us to model treatment effects across the aridity gradient while accounting for the hierarchical structure of the experiment (i.e., repeated application of treatments across aridity levels). We then examined root‐mass fraction separately by modelling sterilisation as a fixed effect and water stress as a random effect, using data subsets for high‐ and low‐aridity soils. Given the limited influence of water stress in these models, we further investigated the interactive effects of sterilisation and aridity using general linear models applied to data subsets representing either control or water‐stressed conditions. Table S2 lists the full fixed and random effect structures and formulae for each model, described above.

We assessed the relative interaction index (RII) ratios for total biomass across the different aridity soils and water stress treatment groups (Armas et al. 2004). For each treatment group, we calculated the average plant response under live and sterilised conditions, using the following formula, where ***x̄*** represents average plant biomass from the live or sterile treatment groups:

$$\mathrm{RII}=\frac{\left(\bar{x}\;\text{Live}-\bar{x}\;\text{Sterile}\right)}{\left(\bar{x}\;\text{Live}+\bar{x}\;\text{Sterile}\right)}$$

Using the R package boot, we generated distributions of the RII values by calculating 95% bias‐corrected and accelerated (BCa) bootstrapped confidence intervals from 10,000 repetitions. Significant differences were found when there was no overlap between the 95% confidence intervals with the mean RII values of other treatments.

#### 2.5.3 Bacterial Diversity Analysis

To understand the distribution of different bacterial ASVs across plant–soil communities, we visualised the relative abundance and the taxonomic occurrence of ASVs within the dominant phyla. To complement relative abundance analyses and provide a secondary validation approach, we calculated phylum‐level ASV occurrence per 1000 ASVs per sample. This method reflects the proportion of ASVs assigned to each phylum, independent of their read abundance. For each sample, we counted the number of ASVs assigned to each phylum and scaled these counts by the total number of present ASVs (i.e., per 1000 ASVs) to standardise across samples with differing richness. We then used differential abundance analysis to evaluate differences across treatments using the *ancombc2* function in the R package ANCOMBC (Lin and Peddada 2020).

To analyse bacterial alpha diversity, we first normalised ASV abundances across all soil, rhizosphere and endosphere samples to account for variation in library sizes. We produced reads‐based and coverage‐based rarefaction curves to visualise how ASV counts changed with sequencing depth (Cameron et al. 2021, Chiu 2023, Schloss 2024; Figure S4a). To ensure that reads‐based rarefaction adequately captured rare taxa, we assessed sample completeness using Good's coverage, which estimates the proportion of total sequences in a sample that belong to ASVs represented more than once. Here, the Good's coverage scores sat at an average of 99.98% with a minimum sample score of 99.92% (Figure S4b,c). Following these evaluations, we proceeded to rarefy all samples to 18,738 reads; because all samples prior to rarefying fell within a Good's coverage range of 99.9%–100%, we did not perform subsequent specialised rarefying or diversity analyses at predefined coverage levels, as per Chiu (2023).

Our alpha diversity calculations across plant compartments and treatments were estimated via ASV richness and the effective number of ASVs by taking the exponential transformation of Shannon's diversity (Jost 2006). Comparisons in alpha diversity levels across treatments were conducted using permuted linear mixed‐effects models and permuted analysis of variance (ANOVAs). Bacterial communities were visualised using nonmetric multidimensional scaling (NMDS) and principal coordinates analysis (PCoA) ordinations with Bray–Curtis dissimilarities (relative abundance) and Jaccard distances (presence/absence). The effect of treatments on the bacterial communities was estimated via permutational multivariate analysis of variance (PERMANOVA) using the *adonis2* function in vegan (Oksanen et al.^2019^ ).

#### 2.5.4 Canonical Correspondence Analysis

To explore how these variables influenced soil microbial communities, we conducted canonical correspondence analysis (CCA). Prior to the CCA, we removed noninformative covariates and selected explanatory variables using the *ordistep2* function from the vegan R package (Oksanen et al. 2019).

## 3 Results

### 3.1 Soil Physicochemical Analysis

Organic carbon and pH were both higher in low‐aridity soils compared with high‐aridity soils. Sterilisation increased ammonium levels in high‐aridity soils, and potassium for low‐aridity soils (Figure S5). Nitrate, phosphorus, sulphur and electrical conductivity did not statistically differ across treatments. At harvest, most soil physicochemical variables showed differences across treatments, except for ammonium, nitrate and electrical conductivity, which did not differ (Figure S5). The greatest differences were increases in phosphorus in sterilised soils, higher potassium in the high‐aridity soils, higher organic carbon in the low‐aridity soils and higher pH in the low‐aridity soils (Figure S5).

### 3.2 Germination and Plant Trait Analysis

We found no effects of high‐/low‐aridity soils (hereafter referred to as soil aridity) or sterilisation on germination rates (GLMM: *z* = 0.422, *p* = 0.673 and *z* = −0.426, *p* = 0.670, respectively; Figure S6). However, larger *T. triandra* seeds germinated earlier than smaller seeds (*z* = 9.97, *p* < 0.001).

When we accounted for soil aridity as a random effect, water stress and soil sterilisation significantly reduced the total *T. triandra* biomass (Figure 1b) compared to control (no‐stress) and live soil conditions (LMM: *t* = −6.877, *p* < 0.001, and *t* = 6.566, *p* < 0.001, respectively). The water stress–sterilisation interaction was also significant here, with the most notable difference being increased biomass in the live control (no‐stress) soil treatment (*t* = −4.183, *p* < 0.001; Table S2, Figure S7). In a separate model, where soil aridity was treated as a fixed effect, and sterilisation and water stress were included as fixed effects and as random intercepts to account for their influence on residual variation, the plants in the live high‐aridity soils produced more biomass than those in live low‐aridity soils (*t* = 6.246, *p* < 0.001). The soil aridity–water stress interaction was also significant, indicating higher plant biomass under high‐aridity soil conditions (*t* = 2.323, *p* = 0.022; Figure S7). We did not detect significant interactions between soil aridity and sterilisation (*t* = −1.015, *p* = 0.311) or between soil aridity, sterilisation and water stress (*t* = −0.910, *p* = 0.364; Table S2, Figure S7).

The aboveground biomass of *T. triandra* plants (Figure 1c) was lower in the water stress (LMM: *t* = 5.700, *p* < 0.001) and sterilisation (*t* = −5.549, *p* < 0.001) when we accounted for soil aridity as a random intercept. Here, a water stress–sterilisation interaction was also present, where aboveground plant biomass was greater under live soil with control water conditions; however, it was unaffected by sterile soil conditions when under both water stress and control conditions (*t* = −3.539, *p* < 0.001; Table S2, Figure S8). Like total biomass, we found higher *T. triandra* aboveground biomass for plants grown under high than low‐aridity soil conditions (*t* = 6.983, *p* < 0.001) when water stress and sterilisation were both included as random intercepts to account for their variation in the models. We also found significant interactions between soil aridity–sterilisation (*t* = −2.221, *p* = 0.032), soil aridity–water stress (*t* = 4.252, *p* < 0.001) and soil aridity–sterilisation–water stress interactions (*t* = −2.046, *p* = 0.046; Figure S8). There was a stronger increase in aboveground biomass in the live high‐aridity soils compared with the sterile high‐aridity soils. We also found that the aboveground biomass increase was greater between the water stress and the control treatments in high‐aridity soils than in the low‐aridity soils (Figure S8).

**FIGURE 2:**
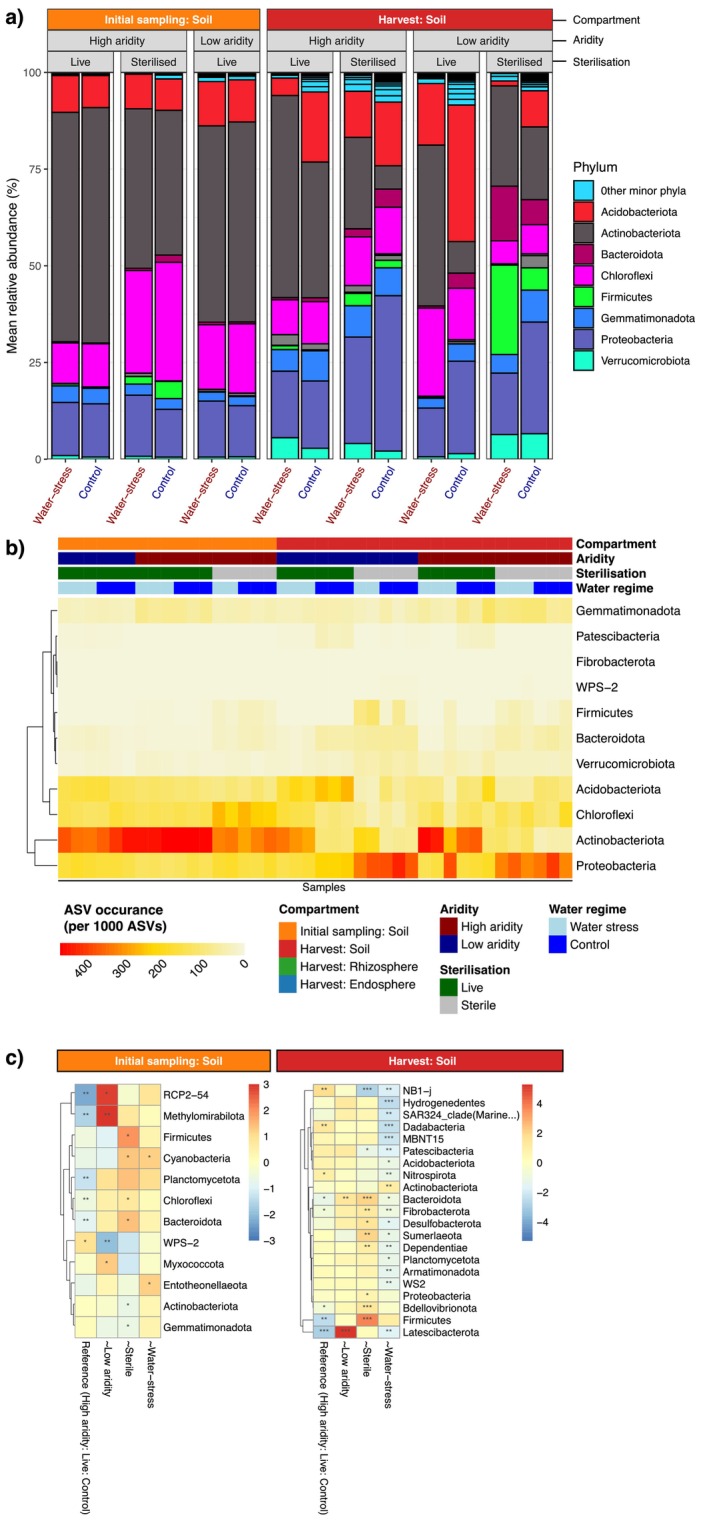
Mean relative abundance of major bacterial phyla across plant‐present pots within *Themeda triandra* compartments over time. (a) Compartment and time point included were the initial soil sampling period, soils at plant harvest, *T. triandra* rhizospheres at plant harvest and *T. triandra* endospheres at plant harvest. Treatments include sterilisation (live, sterile), soil aridity (high, low‐aridity soils) and watering regime (water stress as red text labels, control as blue text labels). We did not sequence viable DNA from sterilised low‐aridity soils. (b) Heatmap of ASV occurrence per 1000 ASVs within each phylum across each sample. Values represent the number of ASVs assigned to each phylum, scaled by the total number of ASVs per sample. Samples are annotated by compartment, water regime, sterilisation treatment and aridity. (c) Differential abundance analysis comparing changes in phyla within each time point and compartment across treatments. Each category compares differences to a reference group (the high aridity, live, control soil treatment). Log fold changes for the reference groups identify differences from the grand mean of each phyla.

Our belowground biomass (Figure 2d) models, when controlling for soil aridity as a random intercept, revealed decreased plant growth when under water stress (LMM: *t* = 3.67, *p* < 0.001) and sterilisation (*t* = −4.864, *p* < 0.001). Furthermore, we also found an interaction between sterilisation and water stress (LMM: *t* = −2.484, *p* = 0.014; Table S2). In our second model, which included sterilisation and water stress as random intercepts to account for variation associated with these treatments, plants grown in high‐aridity soils had significantly greater belowground biomass than those in low‐aridity soils (*t* = 3.586, *p* < 0.001). Here, sterilisation reduced belowground biomass more under low‐aridity soil conditions than under high‐aridity soil conditions. Within this model, however, we did not find interactions between soil aridity and sterilisation (*t* = 0.470, *p* = 0.637; Figure S9), between aridity and water stress (*t* = −0.900, *p* = 0.363) and between aridity, sterilisation and water stress (*t* = 0.834, *p* = 0.403; Table S2).

There was no effect of water stress on root‐mass fraction (Figure 2e). However, in our mixed‐effects models accounting for water stress as a random intercept, sterilisation of low‐aridity soils increased root‐mass fraction (LMM: *p* = 0.003; Table S2), while sterilisation of high‐aridity soils decreased it (*p* = 0.002; Table S2, Figure S10). Additionally, our general linear models using only the control (no water stress) data showed that root‐mass fraction differed significantly between soil aridity levels (LM: *t* = −3.305, *p* = 0.002), between sterilisation treatments (*t* = −3.259, *p* = 0.003), and in their interaction (*t* = 3.527, *p* = 0.001). In contrast, under water stress, there was no difference in root‐mass fraction between high‐ and low‐aridity soils (LM: *t* = −1.664, *p* = 0.104), but significant effects of sterilisation (*t* = −2.303, *p* = 0.026) and its interaction with soil aridity (*t* = 2.860, *p* = 0.007) remained (Table S2, Figure S10).

The RII values were positive for each treatment, though we found significantly higher RII values in the low‐aridity soils compared with high‐aridity soils (see Table S3, Figure 6b). The elevated RII values in low‐aridity soils appear to be driven by the very low biomass outcomes when these soils were sterilised (Figure 1b–d). In the low‐aridity soils, the RII values were higher in the control treatments compared with water stress treatments (Table S3, Figure 6b).

### 3.3 Bacterial Diversity Across Belowground Compartments

We observed 11 bacterial phyla across all samples, which represented 94.8% of reads and had abundance estimates of > 10% across all plant compartments, treatments and time points throughout this experiment (Figure 2a). The soil‐only pots had eight bacterial phyla, which represented 96.9% of reads and had abundance estimates of > 2.5% (Figure S11a). These same bacterial phyla were explored using the taxonomic occurrence of ASVs in the experimental pots and soil‐only pots (Figure 2b and Figure S11b, respectively). Across these analyses, we found notable increases and decreases of bacterial phyla across each of the plant compartments, treatments and time points. In particular, we observed a major increase in ASVs assigned to firmicutes and fibrobacterota in the root endospheres of plants grown in the sterilised soils. We also observed general increases in actinobacteriota in the endospheres, compared with rhizospheres, and increases of proteobacteria and acidobacteriota in the rhizospheres compared with bulk soil levels. However, the strength of these effects did vary depending on the various abundance measurements used (see Figure 2a–c). Soil aridity, sterilisation, and water stress treatments had effects on differential abundance of bacterial phyla across the soils, rhizospheres and endospheres (Figure 2c, Tables S6 and S7). We also found differentially abundant bacterial phyla across each control pot (plant‐absent) across and each treatment (microbial aridity, sterilisation and water stress) (Table S8).

**FIGURE 3:**
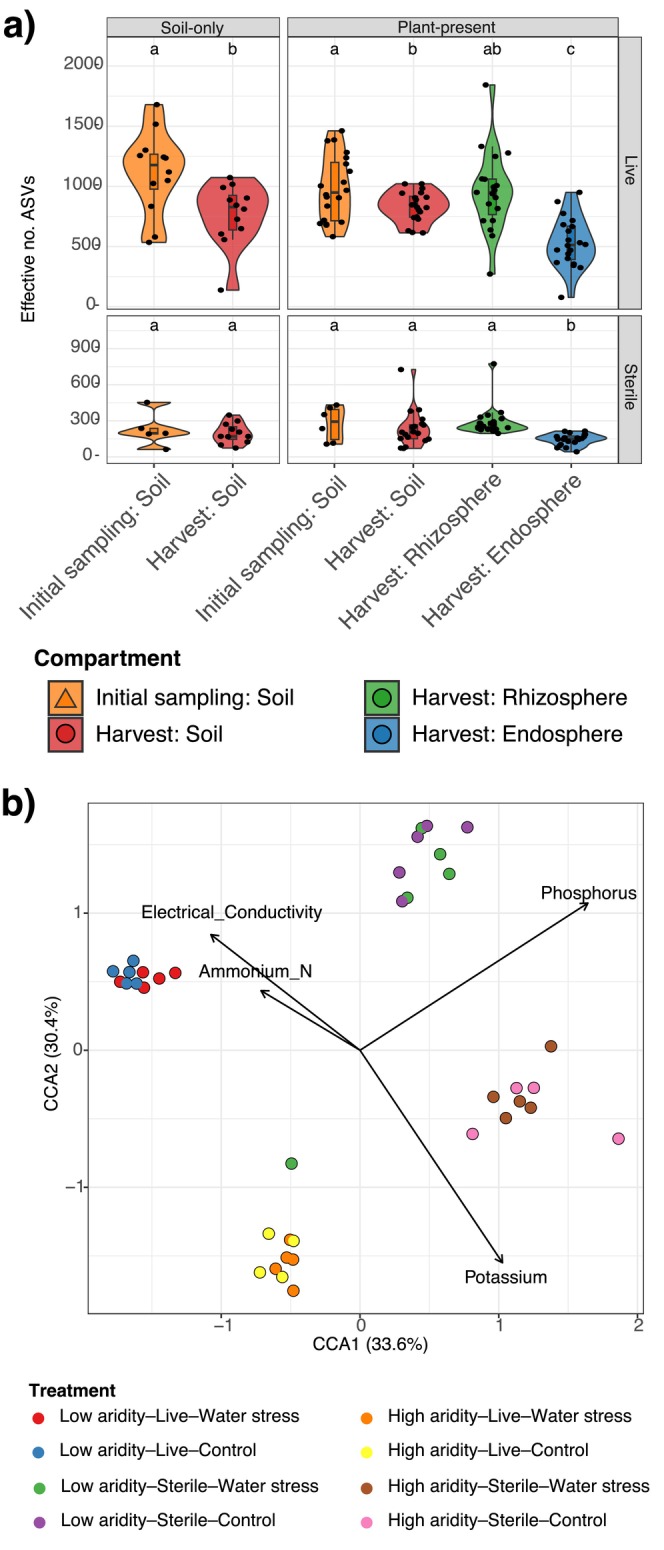
Bacterial alpha diversity differences across *Themeda triandra* compartments and soil physicochemical influences (a) Alpha diversity (effective number of ASVs) across treatments, time and plant‐present versus soil‐only pots. Sample library sizes were rarified to 18,738 reads. Pairwise significance between treatments is indicated by unique letters within each subplot (see Table S5). (b) Canonical correspondence analysis (CCA) of bacterial communities at t1 (plant harvest) compared with significant soil physicochemical variables. Points are coloured by site.

Alpha diversity levels (effective number of ASVs) across the soils and rhizospheres were both higher than the *T. triandra* endospheres in the live (permuted ANOVA: *F*_(3,75)_ = 14.26, *p* < 0.001; Figure 3a) and sterilisation (*F*_(3,63)_ = 5.824, *p* = 0.003). Alpha diversity was also higher for soil‐only pots (in all treatments) at the beginning of the trial than at harvest (*F*_(2,22)_ = 7.932, *p* = 0.01); however, there were no differences between soils in the sterilised soil‐only pots over time (*F*_(1,14)_ = 0.313, *p* = 0.59). Bacterial richness was significantly different among the live plant compartments (*F*_(3,75)_ = 2.765, *p* = 0.049); however, we did not observe any significant pairwise differences after applying FDR corrections (Table S5, Figure S12). In the live control pots, bacterial richness also differed significantly between the initial sampling and harvest soils (*F*_(1,22)_ = 6.029, *p* = 0.022), whereas no such difference was observed in the sterilised control pots (*F*_(1,14)_ = 3.850, *p* = 0.0641).

Sterilisation reduced the effective number of ASVs alpha diversity metric of all soils at the beginning of the experiment (permutedLMEM: *t* = −8.760, *p* < 0.001), and these differences persisted until harvest (*t* = −17.296, *p* < 0.001; Table S6, Figure S13). We saw no effect of soil aridity (live: *t* = −0.156, *p* = 0.875; sterile: *t* = −1.774, *p* = 0.086) or water stress treatment (live: *t* = −1.049, *p* = 0.312, sterile: *t* = 0.211, *p* = 0.833) on soil alpha diversity (Table S6, Figure S13). The soil‐only pots also did not vary in alpha diversity between the live or sterilised soil conditions when accounting for plant compartment, water stress and soil aridity as random intercepts (live: *t* value = −0.567, *p* = 0.584, sterile: *t* value = 1.159, *p* = 0.255). Additionally, sterilisation reduced the richness of the soils at the beginning of the experiment (*t* = −7.294, *p* < 0.001) and these differences persisted until harvest (*t* = −12.680, *p* < 0.001; Table S6, Figure S14). We saw no differences between soil aridity levels (live: *t* = −0.442, *p* = 0.672; sterile: *t* = −1.774, *p* = 0.095) or water stress (live: *t* = −0.948, *p* = 0.358, sterile: *t* = −1.988, *p* = 0.055) on bacterial richness (Table S6, Figure S14). The soil‐only pots also did not vary in richness between the live or sterilised soil conditions when accounting for plant compartment, water stress and soil aridity as random intercepts (live: *t* value = −0.644, *p* = 0.516, sterile: *t* value = 1.171, *p* = 0.257; Table S6).

**FIGURE 4:**
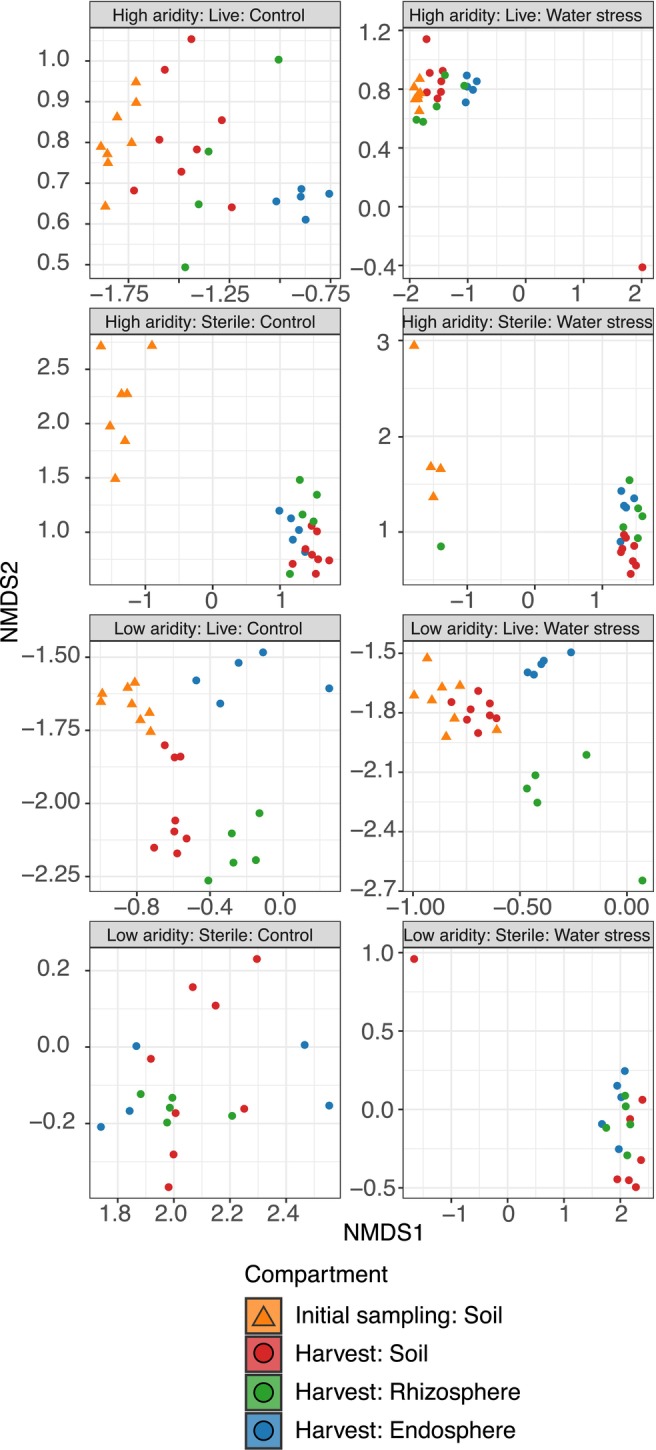
Bacterial community differences across *T. triandra* compartments and time points. Nonmetric multidimensional scaling (NMDS) plot showing bacterial community composition differences for each sampling treatment. Each point represents a sample, and closer points have more similar communities. Sample library sizes were rarified to 18,738 reads.

**FIGURE 5:**
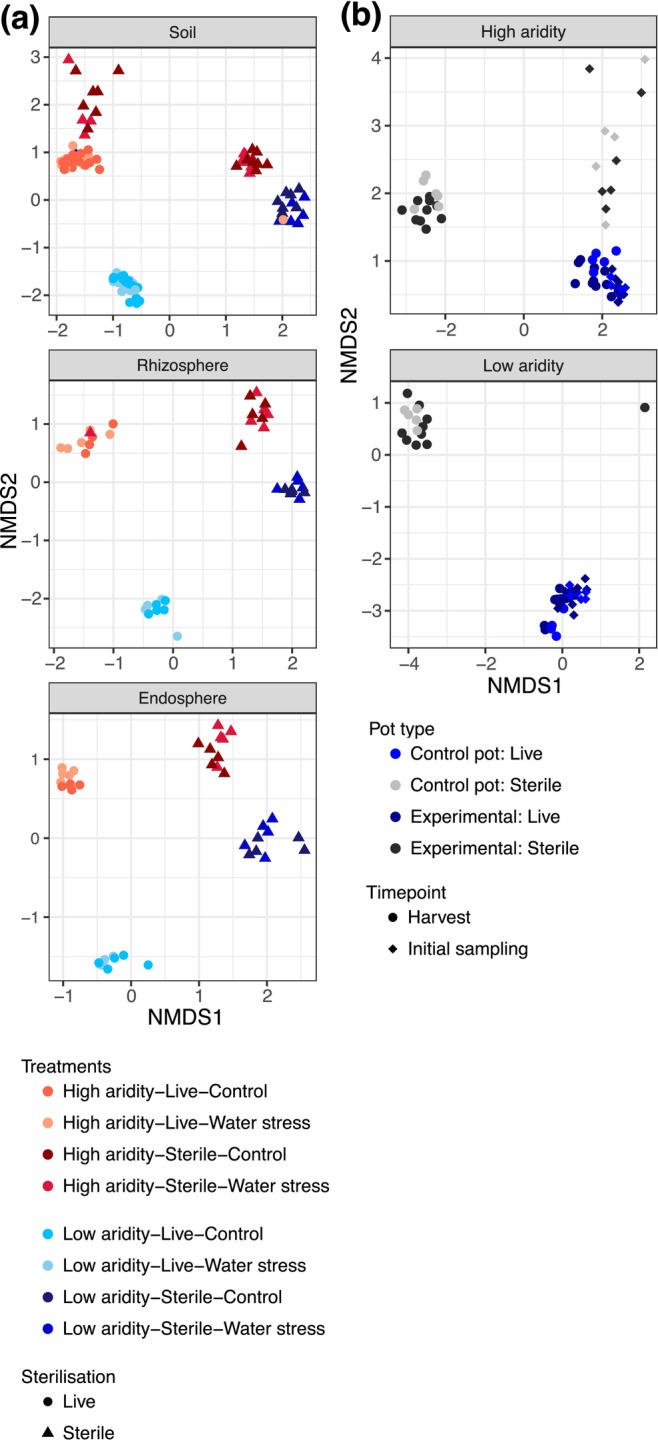
Bacterial community differences using Bray–Curtis dissimilarity across each experimental treatment and comparisons to soil‐only pots. Nonmetric multidimensional scaling (NMDS) plot showing bacterial community composition differences across treatments in (a) sample types from plant‐present pots and (b) soil‐only containing low versus high‐aridity soils. All NMDS ordinations are based on Bray–Curtis distances that emphasise relative abundance of taxa (sample library sizes were rarified to 18,738 reads). Each point represents a sample, and closer points have more similar communities.

Bacterial communities significantly varied by compartment (i.e., soils, rhizospheres, endospheres) across all treatments using Bray–Curtis distances in our NMDS and PCoA ordinations (PERMANOVA: *F*_(2,183)_ = 7.465, *R* ^2^ = 0.075, *p* < 0.001; Figure 4b and Figure S15, respectively). In the soils, bacterial communities differed by sterilisation (*F*_(1,102)=_ 18.117, *R* ^2^ = 0.126, *p* < 0.001), soil aridity (*F*_(1,102)_ = 22.129, *R* ^2^ = 0.153, *p* < 0.001) and water stress treatments (*F*_(1,102)_ = 2.116, *R* ^2^ = 0.015, *p* < 0.013), with these factors collectively explaining 29.4% of the total variance in our PERMANOVAs (Table 1, Figure 4a). For the rhizosphere compartment, communities differed by sterilisation (*F*_(1,36)_ = 11.630, *R* ^2^ = 0.189, *p* < 0.001), soil aridity (*F*_(1,36)_ = 11.737, *R* ^2^ = 0.191, *p* < 0.001) and water stress treatments (*F*_(1,102)_ = 2.215, *R* ^2^ = 0.036, *p* = 0.023), with these factors collectively explaining 42.5% of the total variance (Table 1, Figure 4a). Finally, for the endosphere compartment, communities differed by sterilisation (*F*_(1,36)_ = 12.696, *R* ^2^ = 0.207, *p* < 0.001), soil aridity (*F*_(1,36)_ = 10.522, *R* ^2^ = 0.172, *p* < 0.001) and water stress treatments (*F*_(1,36)_ = 1.976, *R* ^2^ = 0.032, *p* = 0.033), with these factors collectively explaining 41.1% of the total variance (Table 1, Figure 4a). The soil‐only pots did not differ from the plant‐present pots (*F*_(1,103)_ = 0.733, *R* ^2^ = 0.007, *p* = 0.755), but the bacterial communities did differ across time points (*F*_(1,103)_ = 0.733, *R* ^2^ = 0.007, *p* = 0.755; Figure 5).

**TABLE 1:** Statistical output for alpha beta diversity (bacterial community composition) across *Themeda triandra* bulk soils, rhizospheres and endospheres using permuted multivariate analysis of variance (PERMANOVA).

Distance metric	Compartment	Predictor variable	Degrees of freedom	*F*‐statistic	*R* squared	*p*
Bray–Curtis	Soil	Aridity	1 and 102	22.129	0.153	*p* < 0.001***
		Sterilisation	1 and 102	18.117	0.126	*p* < 0.001***
		Water Stress	1 and 102	2.116	0.015	*p* = 0.013*
	Rhizosphere	Aridity	1 and 36	11.737	0.191	*p* < 0.001***
		Sterilisation	1 and 36	11.630	0.189	*p* < 0.001***
		Water Stress	1 and 36	2.215	0.036	*p* = 0.023*
	Endosphere	Aridity	1 and 36	10.522	0.172	*p* < 0.001***
		Sterilisation	1 and 36	12.696	0.207	*p* < 0.001***
		Water Stress	1 and 36	1.976	0.032	*p* = 0.033*
Jaccard	Soil	Aridity	1 and 102	8.011	0.068	*p* < 0.001***
		Sterilisation	1 and 102	6.315	0.054	*p* < 0.001***
		Water Stress	1 and 102	1.430	0.012	*p* = 0.033*
	Rhizosphere	Aridity	1 and 36	4.094	0.090	*p* < 0.001***
		Sterilisation	1 and 36	3.990	0.087	*p* < 0.001***
		Water Stress	1 and 36	1.522	0.033	*p* = 0.023*
	Endosphere	Aridity	1 and 36	4.262	0.094	*p* < 0.001***
		Sterilisation	1 and 36	3.846	0.084	*p* < 0.001***
		Water Stress	1 and 36	1.406	0.031	*p* = 0.032*
Bray–Curtis	Soil	Time point	1 and 103	7.354	0.066	*p* < 0.001**
		Plant presence	1 and 103	0.733	0.007	*p* = 0.755
Jaccard	Soil	Time point	1 and 103	3.603	0.034	*p* < 0.001***
		Plant presence	1 and 103	0.935	0.009	*p* = 0.582

*Note:* PERMANOVAS for the soil, rhizospheres and endospheres were calculated using the *adonis2* function in vegan. *R* squared includes the unique proportion of variation explained by each predictor (i.e., the partial *R* squared). Total variation explained per compartment in the Bray–Curtis distance models is approximately 29.4% for soil, 42.5% for rhizosphere and 41.1% for endosphere. For the Jaccard distance models, total variation explained per compartment is approximately 13.2% for soil, 21.1% for rhizosphere and 20.9% for endosphere. *p values < 0.05, **p values < 0.01, ***p values < 0.001.

Bacterial communities using Jaccard distances significantly varied by compartment (i.e., soils, rhizospheres, endospheres) in our NMDS and PCoA ordinations (PERMANOVA: *F*_(2,183)_ = 3.610, *R* ^2^ = 0.038, *p* < 0.001; Figure 4 and Figure S16, respectively). Overall, we found the same significant results for our bacterial communities using Jaccard distances in our NMDS and PCoA ordinations (see Table 1 for more detail; Figures S16 and S17, respectively).

There was no detectable difference between the bacterial community compositions between the soil‐only pots and the plant‐present pots (PERMANOVA: *F*_(1,103)_ = 0.733, *R* ^2^ = 0.007, *p* = 0.755; Figure 4b), but we did observe a difference in communities from the initial sampling to the harvest (PERMANOVA: *F*_(1,103)_ = 7.354, *R* ^2^ = 0.066, *p* < 0.001; Figure 4b, Figure S18).

### 3.4 Plant–Microbial Diversity Trends

**FIGURE 6:**
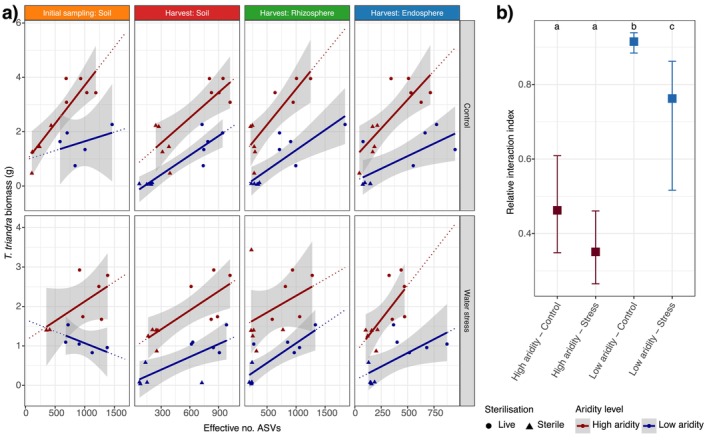
Bacterial diversity is correlated with *Themeda triandra* total biomass. (a) Alpha diversity (effective number of ASVs) is positively correlated with postharvest *T. triandra* biomass across all plant compartments and watering treatments. Soil aridity is denoted by colour (red = high‐aridity soils, blue = low‐aridity soils), and soils exposed to sterilisation at the beginning of the trial are shown with point shape (sterilisation = triangles, live = circles). (b) Relative Interaction Index (RII) for *T. triandra* plants grown under water stress and control conditions, and in soils from high‐ and low‐aridity soils. The RII ranges from −1 to 1 and reflects the net effect of soil microbial communities on plant growth. Values > 0 indicate a beneficial effect of live microbial communities compared to sterilised soils, while values < 0 indicate improved growth in the absence of live microbes. Squares represent treatment means, and error bars indicate 95% confidence intervals. Pairwise significance between treatments is indicated by unique letters within each subplot (see Table S3).

Bacterial alpha diversity in soils, rhizospheres and endospheres correlated positively and strongly with plant biomass (LMEM: *t* value = 10.857, *p* < 0.001; Figure 6a). Biomass increased more with bacterial alpha diversity in high‐aridity soils (Figure 6a), but also with bacterial richness (*t* value = 9.068, *p* < 0.001) (Figure S19).

### 3.5 Canonical Correspondence Analysis

Our CCAs found that phosphorus and potassium were associated with the highest level of bacterial community change, followed by electrical conductivity and nitrate nitrogen levels. The overall CCA model was significant (*F*_(4,33)_ = 1.94, *p* < 0.001, 999 permutations). There was also clustering of bacterial communities into four distinct groups based on each of the live‐sterile and high‐low soil aridity levels, though there was no clear difference between the communities of water stress and control plants (Figure 3b). Notably, we excluded from the CCA model the variables of pH and organic carbon, which both covaried with potassium, and sulphur, which covaried with electrical conductivity (*r* > 0.75) (Figure S20).

## 4 Discussion

We experimentally assessed the effects of high and low‐aridity soil legacies on the growth of the keystone grass species, *Themeda triandra* , under drought conditions. We show that microbiota from high‐aridity soils supported increased growth of this grass species under both drought‐like water stress and control treatments, highlighting the powerful impact of soil legacies and supporting our first hypothesis. We also show that bacterial alpha diversity was positively correlated with *T. triandra* biomass, and that each of our treatments (i.e., soil aridity, sterilisation and water stress) led to distinct bacterial assemblages in soils, rhizospheres and endospheres. This supports our second hypothesis by highlighting the conditional relationships that *T. triandra* forms with its bacterial communities that are dependent on its environment. We also found key bacterial phyla in root endospheres that were strongly linked to seed endophytes, suggesting vertical transmission of microbiota (i.e., parent‐to‐offspring inheritance during seed development). Finally, we did not see meaningful differences across the bacterial communities of our soil‐only versus plant‐present pots, which goes against the expectations of our third hypothesis. Our findings highlight the importance of soil microbiota for host plant growth and fitness under climate change. Our study underscores the importance of protecting diverse soil communities to support grassland health and highlights the potential of harnessing these communities to increase grassland restoration that is more resilient to climate change.

### 4.1 Microbially Mediated Effects on Biomass

We show that live soil communities enhanced plant growth in high‐ and low‐aridity soils, and under control and water‐stress conditions. Additionally, bacterial alpha diversity across the soils, rhizospheres and endospheres was positively correlated with *T. triandra* biomass, suggesting that a greater variety of unique bacteria, either naturally present in the soil or recruited into the rhizospheres and endospheres, leads to greater plant growth. Alpha diversity is a well‐known driver of plant productivity and is associated with greater ecosystem functionality (Schnitzer et al. 2011; Byrnes et al. 2014; Wang et al. 2019). Our findings support previous research that shows that host‐benefiting microbial functions are present within *T. triandra* soils, rhizospheres and endospheres (Hodgson et al. 2024), and the importance of the habitat source of microbes (e.g., arbuscular mycorrhizal fungi) for *T. triandra* drought response (Petipas et al. 2017). As such, there is now a strong body of evidence to suggest that soil microbiota support *T. triandra* growth across diverse ecosystems, under both stress and nonstress conditions.

Our *T. triandra* plants developed larger root‐mass fractions in the sterilised high‐aridity soils, compared with the live high‐aridity soils. This shows that a higher proportion of plant resources were allocated to the development of roots under sterilised soil conditions, perhaps in response to an absence of microbiota which typically aid the acquisition or unblocking of nutrient resources in the soil (Pérez‐Harguindeguy et al. 2013; Bai et al. 2022; Wang et al. 2024). Interestingly, we observed the opposite trend in the low‐aridity soils, where greater root investment occurred in the live low‐aridity soils compared to those grown in sterilised low‐aridity soils. As the bacterial communities were distinct, the low‐aridity soil microbiota may not provide the same functional benefits as those found in the high‐aridity soils—where different soil conditions, such as available moisture or organic matter, could create different host needs (Hodgson et al. 2024). Plants growing in the low‐aridity soils may not typically produce such strong microbial‐root interactions, given the potential absence of these stress–tolerance benefits by the microbiota (Comas et al. 2013; Hodgson et al. 2024). The importance of microbiota for plant growth, and the strength of the interaction they form, may therefore depend on the aridity of the soil and plant growth environment (De Long et al. 2019).

### 4.2 Treatment Effects on the Two‐Step Selection Process

We show a decrease in bacterial diversity in the rhizosphere and endosphere versus bulk soils, which is consistent with previous findings expected under the two‐step selection process (Bulgarelli et al. 2012; Lundberg et al. 2012; Urbina et al. 2018). *T. triandra* plants recruited different communities of bacteria from the soil into their rhizospheres and endospheres depending on whether they underwent soil sterilisation or water stress treatments. These findings suggest that the plant's growth environment alters the recruitment dynamics of soil bacteria. They also indicate that *T. triandra* plants appear to alter their entry screening strategies of soil bacteria when growing under drought‐like conditions. However, these microbial communities may also be shaped by competitive exclusion processes among bacterial taxa, which can alter community structure in response to differing environments and available resources, such as those found in the rhizosphere and endosphere.

Endosphere recruitment dynamics were most sensitive to the effects of soil sterilisation in this experiment, compared to soil aridity or water stress. In all sterilised soils, endosphere diversity was lower and bacterial communities were differently structured to the unsterilised soils. Notably, firmicutes were highly abundant in sterilised endosphere treatments and were almost absent from unsterilised ones. This phylum includes many known seed‐associated bacteria (Johnstone‐Monje et al. 2021), suggesting that these taxa may have originated from seed microbiota and spread into root tissues via vertical transmission—the passage of bacteria from parent to offspring (Truyens et al. 2015). The low bacterial diversity levels in the endospheres grown in sterilised soil conditions, combined with the absence of key phyla found in live root communities and the distinct beta‐diversity profiles (community compositions), suggest that vertically transmitted taxa may have filled the endosphere niche in the absence of competition from typically soil‐selected microbes. Alternatively, the reduced *T. triandra* growth rates in the low‐aridity sterilised soils (their biomass was lower than in high‐aridity sterilised soil treatments) may indicate that the endospheres were more susceptible to colonisation by opportunistic pathogens, which can proliferate in the low‐competition environment created by soil sterilisation (Mallon et al. 2015; Mawarda et al. 2022).

In summary, our experiment cannot confirm whether endosphere bacteria in sterilised soils were remnants of incomplete sterilisation and/or originated from the seed microbiome (Kim et al. 2022; Ling et al. 2022; Abdelfattah et al. 2023; He et al. 2024). Further validation will be essential to answer this question, and shotgun metagenomic analysis will help identify properties of microbial endosphere colonisation, such as the acquisition of growth‐promoting functions. This could help reveal whether colonisation dynamics are being hijacked by pathogenic or opportunistic microbes (i.e., ‘cheater’ organisms) that do not provide the same host plant services, despite other shared traits (Kiers et al. 2002, 2011). The consequences of these interactions could help inform the vulnerability of *T. triandra* to soil degradation, making this an important avenue for future research.

### 4.3 Soil Physicochemical Variation

Soil physicochemical properties were significant predictors of bacterial community variation, with factors such as electrical conductivity and concentrations of ammonium, phosphorus, and potassium associated with community composition. These variables also differed across treatments, particularly between high‐ and low‐aridity soils, suggesting they could influence experimental outcomes. For example, variation in organic carbon, pH, and potassium may have shaped how soils responded to autoclaving (Berns et al. 2008). Further work to better standardise soil conditions, or to more clearly disentangle plant–microbial interactions, would help isolate the specific effects of microbial communities.

## 5 Conclusion

In our study, we sought to determine whether the widespread keystone grass, *T. triandra*, relied on soil microbiota from arid locations to gain growth advantages when grown under drought conditions. We report that soil microbiota from more arid sources had strong positive effects on plant growth under drought conditions. We also show that soil aridity, water stress and sterilised treatments shaped both plant growth and the soil‐to‐endosphere recruitment as described by the two‐step selection process. Finally, soil physicochemical variables associated with our stress and sterilisation conditions influenced the composition of bacterial communities far more strongly than the presence of *T. triandra* plants. Together, these results highlight that grassland decline by increased aridity due to climate change may be in part mitigated by the effects of healthy soil microbiota on keystone grass species. As such, we anticipate that soil‐based manipulations to enhance the restoration of climate‐resilient grasslands will be increasingly applicable to future interventions in grassland ecosystems.

## Author Contributions

Project design: R.J.H., S.A.R., M.F.B. Field work: R.J.H., S.D.P., D.S. Laboratory work: R.J.H., C.C.‐D., T.D., V.D., N.W.F., A.T. Statistical analysis, writing manuscript: R.J.H. All authors revised and edited the manuscript and gave approvals before final submission.

## Disclosure

Benefit Sharing: Benefits from this research accrue from the sharing of our data and results on public databases, as described above.

## Conflicts of Interest

The authors declare no conflicts of interest.

## Supporting information


**Data S1:** mec70062‐sup‐0001‐DataS1.docx.

## Data Availability

Data and code supporting this analysis can be found at DOI 10.25451/flinders.27957138
