# Supplementary material for: Arid Soil Bacterial Legacies Improve Drought Resilience of the Keystone Grass, Themeda triandra
Source: Mol Ecol. 2025 Aug 18;34(18):e70062. doi: 10.1111/mec.70062 (PMC12421485; doi:10.1111/mec.70062)
Supplement: Supplementary file 1 — Data S1: mec70062‐sup‐0001‐DataS1.docx. [file MEC-34-e70062-s001.docx]

# ***Supplementary Information for***

# **Arid soil bacterial legacies improve drought resilience of the keystone grass, *Themeda triandra***

**Figure S1. Experimental design for *T. triandra* growth trial.** a) 8 experimental treatments with microbiota sourced from high (sun) and low aridity (cloud) soils, plants are then raised in these soils under live (block colour) and sterilised (striped colour) conditions, then subjected to water stress and control treatments (10 pots per treatment). (b) After sowing of 8 plant seeds per pot, and thinning of seedlings (one per pot), plants across each treatment are grown in the glasshouse for a total of 5 months, before growth trait measurements are made during harvest. (c) An additional 24 soil-only pots were maintained across these treatments with not T. triandra seeds planted to monitor changes in microbial communities in the absence of growing plants (8 treatments, 3 pots per treatment).

**Figure S2. The relative soil water content (RSWC) percentage of each pot throughout the duration of the growth trial.** Each line represents a single pot, coloured by treatment group including label codes for: High (Q) and low (K) microbial aridity; live (L) and sterile (S)treatments; water stress (D) and control (W), as well as the plant absent, control pots (C). Dotted lines indicate watering levels throughout the experiment. Control treatments were watered to 100% RSWC throughout the duration, whereas the water stress treatments were gradually stressed first to 70%, then 50% and finally 40% RSWC. RSWC is based on the average of lowest RSWC value (before watering) and highest RSWC value (after watering), for each recorded day.

**Figure S3. Seed planting template for pots during germination trial.** Eight different seeds were planted radially with identifying marks on the lip of each part for traceability.

**Figure S4. Reads-based rarefaction of sequences with coverage estimates.** (a) Rarefaction plot showing the number of species (ASVs) against number of reads. (b) Good’s coverage of sequence counts by sequences per sample. Each sample is coloured by plant compartment (soil, rhizosphere, and endosphere) at the initial soil sampling and the plant harvest. Samples were rarefied to 18,738 reads (black vertical lines). (c) the number of species (ASVs) against Good’s coverage of sequence counts.

**Figure S5. Soil physicochemical differences across each plant treatment**. Soil variables included (a) ammonium nitrogen, (b) nitrate nitrogen, (c) phosphorus, (d) potassium, (e) sulphur, (f) organic carbon, (g) electrical conductivity, and (h) pH (CaCl_2_). Each point represents a sampled pot, coloured by treatment group. Treatment codes label codes are described by lettering with: High (Q) and low (K) microbial aridity; live (L) and sterile (S) treatments; water stress (D) and control (W), as well as the plant absent, control pots (C). Unique letters indicates significant differences between treatments at the 0.05 significance level using a Dunn test with Holm adjusted p-values.

**Figure S6. Cumulative germination curve showing average germination per pot, across each soil treatment.** Treatments include: (Q: Quorn, K Kuitpo), and sterilisation treatment (L: Live, S: Sterile). Errorbars represent 95% confidence intervals.

**Figure S7. Interaction plot for *T. triandra* total biomass (g).** Interaction plots show: a) low and high ardity microbiotas, showing how sterilisation impacts *T. triandra* growth under water stress versus control, and b) how low and high ardity microbiotas impact *T. triandra* growth under water stress conditions.

**Figure S8. Interaction plot for aboveground *T. triandra* biomass (g).** a) low and high ardity microbiotas, showing how sterilisation impacts *T. triandra* aboveground biomass under water stress versus control, and b) how low and high ardity microbiotas impact *T. triandra* aboveground biomass under water stress conditions, and c) *T. triandra* aboveground biomass response to water stress is impacted by microbial aridity across live and sterile conditions.

**Figure S9. Interaction plot for belowground *T. triandra* biomass (g)**. a) low and high ardity microbiotas, showing how sterilisation impacts *T. triandra* belogground biomass under water stress.

**Figure S10. Interaction plot for *T. triandra* biomass root-mass fractions.** This plot shows how sterilisation impacts *T. triandra* root investment across low and high aridty microbiotas.

**Figure S11. Mean relative abundance of major bacterial phyla across plant-absent control pots within *T. triandra* pot soils over time.** (a) Compartment and timpeoint included were the initial soil sampling period (t_0_: orange crosslabel), soils at plant harvest (t_1_: red crosslabel). Treatmens include sterilisation (live, sterile), microbiome aridity (high, low arid sourced soil microbiotas), and watering regime (water-stress as red text labels, control as blue text labels). (b) Heatmap of ASV occurrence per 1000 ASVs within each phylum across each sample. Values represent the number of ASVs assigned to each phylum, scaled by the total number of ASVs per sample. Samples are annotated by compartment, water regime, sterilisation treatment, and aridity. (c) Differential abundance analysis comparing changes in phyla within each timpoint and compartment across pot soils. Each category compares differences to a reference group (the high aridity, live, control treatment). Log fold changes for the reference groups show changes compared to the grandmean of each phyla.

**Figure S12. Bacterial richness differences across *T. triandra* compartments, and soil physicochemical influences** (a) bacterial ASV richness across treatments, time, and plant-present versus soil-only pots. Sample library sizes were rarified to 18,738 reads. Compact letter display text shows how pairwise comparisons differ across treatments at the 0.05 significance levels using uncorrected (blue) and corrected comparisons using the false discovery rate (red).

**Figure S13. Bacterial alpha diversity differences across *T. triandra* compartments and timpoints.** Alpha diversity is indicated by the effective number of ASVs for high and low aridity soils, live and sterilised soils, and water stress and control plants.

**Figure S14. Bacterial ASV richness differences across *T. triandra* compartments and timpoints.** ASV richness is presented for for high and low aridity soils, live and sterilised soils, and water stress and control plants.

**Figure S15.** **Principal co-ordinates analysis of bacterial communities associated with *T. triandra* bulk soils rhizospheres and endosphere using Bray-Curtis distances.** These orination plots show patterns of soil to endosphere community colonisation as per the two- step selection process (beta diversity) based on the relative abundance differences of taxa. Axis 1 and 2 refer to the principal components 1 and 2, respectively.

**Figure S16. Bacterial community differences using Jaccard distances across *T. triandra* compartments and timpoints** Non metric multidimenional scaling (NMDS) plot showing bacterial community composition differences for each sampling treatment, based on presence/absence of taxa. Each point represents a sample, and closer points have more similar communities. Sample library sizes were rarified to 18,738 reads.

**Figure S17.** **Principal co-ordinates analysis of bacterial communities associated with *T. triandra* bulk soils rhizospheres and endosphere using Jaccard distances.** These orination plots show patterns of soil to endosphere community colonisation as per the two-step selection process (beta diversity) based on the presence/absence differences of taxa. Axis 1 and 2 refer to the principal components 1 and 2, respectively.

**Figure S18. Bacterial community differences using Jaccard distances across each experimental treatment, and comparisions to soil-only pots.** Non metric multidimensional scaling (NMDS) plot showing bacterial community composition differences across treatments in (a) sample types from plant-present pots, and (b) soil-only containing low versus high aridity soils. All NMDS ordinations are based on Jaccard distances that emphasise presence/absence of taxa (sample library sizes were rarified to 18,738 reads). Each point represents a sample, and closer points have more similar communities.

**Figure S19. Bacterial richness is correlated with *T. triandra* total biomass.** Bacterial richness is positively correlated with post harvest *T. triandra* biomass across all plant compartments, and watering treatments. Soil aridity is denoted by colour (red = high aridity soils, blue = low aridity soils), and soils exposed to sterilisation at the beginning of the trial are shown with point shape (sterilisation = triangles, live = circles).

**Figure S20. Correlation plot showing covariation among soil physicochemical variables.** Variables are displayed in a symmetrical matrix based on Pearson’s correlation coefficients, following standardisation. The size and colour intensity of circles indicate the strength of correlations: blue for positive and red for negative. Where |r| > 0.75, one or more of the correlated variables were removed from downstream analyses to reduce multicollinearity.

**TABLES**

**Table S1. Sampling sites for high and low aridity soil microbiota**. Aridity index data values were extracted from the mean annual aridity index data layer (ADM) and annual precipitation data layer (Clim_PTA) were sourced from the Soil and Landscape Grid of Australia (Searle *et al.* 2022), where aridity index is calculated via annual precipitation/annual potential evaporation.

| **Sampling site** | **Latitude, Longitude** | **Mean annual aridity index** | **Aridity category** | **Annual precipitation (mm)** | **Provenance** |
| --- | --- | --- | --- | --- | --- |
| Quorn Floral Reserve | 32.3434°S, 138.0182°E | 0.227 | High aridity | 355.2 | Soil microbiota |
| Kuitpo Forest Reserve | 35.2279°S, 138.7199°E | 0.658 | Low aridity | 850.5 | Soil microbiota and *T. triandra* seed |

**Table S2.** ***T. triandra* growth traits analysis using randomised linear mixed effects models, with statistical output.** ‘*’ denotes p values <0.05, ‘**’ denotes p values <0.01, ‘***’ denotes p values <0.001. †Only output related to aridity variables is reported.

| **Formula (response and predictors)** | **Random effects** | **Permutations** | **Fixed effects** | **t-statistic** | **P-Value** |
| --- | --- | --- | --- | --- | --- |
| Total biomass ~ Sterilisation x Water stress | Aridity | 10,000 | Sterilisation | -6.877 | <0.001*** |
|  |  |  | Water stress | 6.566 | <0.001*** |
|  |  |  | Sterilisation x Water stress | -4.183 | <0.001*** |
| Total biomass~ Aridity† x Sterilisation x Water stress | Sterilisation + Water stress | 10,000 | Aridity | 6.246 | <0.001*** |
|  |  |  | Aridity x Sterilisation | -1.015 | 0.311 |
|  |  |  | Aridity x Water stress | 2.323 | 0.022* |
|  |  |  | Aridity x Sterilisation x Water stress | -0.910 | 0.364 |
| Aboveground biomass ~ Sterilisation x Water stress | Aridity | 10,000 | Sterilisation | -5.549 | <0.001*** |
|  |  |  | Water stress | 5.700 | <0.001*** |
|  |  |  | Sterilisation x Water stress | -3.539 | <0.001*** |
| Aboveground biomass~ Aridity† x Sterilisation x Water stress | Sterilisation + Water stress | 10,000 | Aridity | 6.983 | <0.001*** |
|  |  |  | Aridity x Sterilisation | -2.221 | 0.032* |
|  |  |  | Aridity x Water stress | 4.252 | <0.001*** |
|  |  |  | Aridity x Sterilisation x Water stress | -2.046 | 0.046* |
| Belowground biomass ~ Sterilisation x Water stress | Aridity | 10,000 | Sterilisation | -4.864 | <0.001*** |
|  |  |  | Water stress | 3.67 | <0.001*** |
|  |  |  | Sterilisation x Water stress | -2.484 | 0.014* |
| Belowground biomass~ Aridity† x Sterilisation x Water stress | Sterilisation + Water stress | 10,000 | Aridity | 3.586 | <0.001*** |
|  |  |  | Aridity x Sterilisation | 0.470 | 0.637 |
|  |  |  | Aridity x Water stress | -0.900 | 0.363 |
|  |  |  | Aridity x Sterilisation x Water stress | 0.834 | 0.403 |
| Root-mass fraction ~ Sterilisation (High aridity only) | Water | 10,000 | Sterilisation | 3.239 | 0.003** |
| Root-mass fraction ~ Sterilisation (Low aridity only) | Water | 10,000 | Sterilisation | -3.373 | 0.0016** |
| Root-mass fraction ~ Aridity x Sterilisation (Control soils only) | NA (General linear model) | 10,000 | Aridity | -3.305 | 0.0016** |
|  |  |  | Sterilisation | -3.259 | 0.0026** |
|  |  |  | Aridity x Sterilisation | 3.527 | 0.001** |
| Root-mass fraction ~ Aridity x Sterilisation (Water stress soils) | NA (General linear model) | 10,000 | Aridity | -1.664 | 0.104 |
|  |  |  | Sterilisation | -2.303 | 0.026* |
|  |  |  | Aridity x Sterilisation | 2.860 | 0.0071** |
| Root-mass fraction ~ Aridity† x Sterilisation x Water stress | Sterilisation + Water stress | 10,000 | Aridity | 3.586 | <0.001*** |
|  |  |  | Aridity x Sterilisation | 0.470 | 0.6368 |
|  |  |  | Aridity x Water stress | -0.900 | 0.3627 |
|  |  |  | Aridity x Sterilisation x Water stress | 0.834 | 0.4033 |

**Table S3. Statistical output for *T. triandra* relative interaction index (RII) comparisons based on biomass with bootstrapped 95% confidence intervals at 10,000 permutations**. Significant differences between pairwise groups, indicated by ‘*’, were interpreted when upper or lower confidence intervals did not alight with means in the comparative treatment.

| **Comparison group 1** | **Comparison group 2** | **Mean difference** | **Lower CI** | **Upper CI** | **Significance** |
| --- | --- | --- | --- | --- | --- |
| High aridity-Stress | Low aridity-Stress | -6.264 | -13.136 | -1.740 | * |
| High aridity-Stress | High aridity-Control | -0.688 | -1.970 | 0.262 |  |
| High aridity-Stress | Low aridity-Control | -21.138 | -29.971 | -14.546 | * |
| Low aridity-Stress | High aridity-Control | 5.576 | 0.927 | 12.424 | * |
| Low aridity-Stress | Low aridity-Control | -14.874 | -25.247 | -5.189 | * |
| High aridity-Control | Low aridity-Control | -20.450 | -29.328 | -13.754 | * |
| High aridity-Control | Low aridity-Control | -38.867 | -60.890 | -22.576 | * |

**Table S4. Summary of sequencing depth and diversity metrics for each sample.** Compartment and Treatment describe the experimental setup. Total Reads is the number of sequences per sample before rarefaction. No. ASVs is the number of unique ASVs detected, and No. ASVs (rarefied) is after rarefaction to 18,738 reads. Good’s coverage (%) estimates sampling completeness.

| **Sample** | **Compartment** | **Treatment** | **Total Reads** | **No. ASVs** | **No. ASVs (rarefied)** | **Good's coverage (%)** |
| --- | --- | --- | --- | --- | --- | --- |
| HEKLD1 | Harvest: Endosphere | Low aridity-Live-Water stress | 131814 | 2716 | 2308 | 99.99 |
| HEKLD3 | Harvest: Endosphere | Low aridity-Live-Water stress | 34176 | 1030 | 1019 | 99.99 |
| HEKLD5 | Harvest: Endosphere | Low aridity-Live-Water stress | 37851 | 1126 | 1095 | 99.97 |
| HEKLD7 | Harvest: Endosphere | Low aridity-Live-Water stress | 58100 | 1568 | 1483 | 99.98 |
| HEKLD9 | Harvest: Endosphere | Low aridity-Live-Water stress | 65197 | 1810 | 1690 | 99.98 |
| HEKLW1 | Harvest: Endosphere | Low aridity-Live-Control | 56937 | 909 | 866 | 100.00 |
| HEKLW3 | Harvest: Endosphere | Low aridity-Live-Control | 64226 | 1600 | 1510 | 100.00 |
| HEKLW5 | Harvest: Endosphere | Low aridity-Live-Control | 50360 | 1558 | 1503 | 99.99 |
| HEKLW7 | Harvest: Endosphere | Low aridity-Live-Control | 118186 | 2586 | 2274 | 99.98 |
| HEKLW9 | Harvest: Endosphere | Low aridity-Live-Control | 35019 | 1214 | 1182 | 99.97 |
| HEKSD2 | Harvest: Endosphere | Low aridity-Sterile-Water stress | 59309 | 613 | 582 | 100.00 |
| HEKSD3 | Harvest: Endosphere | Low aridity-Sterile-Water stress | 83208 | 667 | 620 | 100.00 |
| HEKSD5 | Harvest: Endosphere | Low aridity-Sterile-Water stress | 68760 | 611 | 574 | 99.99 |
| HEKSD7 | Harvest: Endosphere | Low aridity-Sterile-Water stress | 51617 | 568 | 550 | 100.00 |
| HEKSD9 | Harvest: Endosphere | Low aridity-Sterile-Water stress | 1064856 | 1317 | 895 | 100.00 |
| HEKSW1 | Harvest: Endosphere | Low aridity-Sterile-Control | 36064 | 315 | 309 | 100.00 |
| HEKSW3 | Harvest: Endosphere | Low aridity-Sterile-Control | 54558 | 457 | 438 | 99.99 |
| HEKSW5 | Harvest: Endosphere | Low aridity-Sterile-Control | 124666 | 786 | 709 | 100.00 |
| HEKSW7 | Harvest: Endosphere | Low aridity-Sterile-Control | 88645 | 776 | 713 | 100.00 |
| HEKSW9 | Harvest: Endosphere | Low aridity-Sterile-Control | 756447 | 1445 | 913 | 100.00 |
| HEQLD1 | Harvest: Endosphere | High aridity-Live-Water stress | 41203 | 1101 | 1088 | 99.99 |
| HEQLD3 | Harvest: Endosphere | High aridity-Live-Water stress | 64426 | 1415 | 1337 | 99.99 |
| HEQLD5 | Harvest: Endosphere | High aridity-Live-Water stress | 38684 | 971 | 950 | 99.99 |
| HEQLD7 | Harvest: Endosphere | High aridity-Live-Water stress | 32052 | 968 | 961 | 99.98 |
| HEQLD9 | Harvest: Endosphere | High aridity-Live-Water stress | 31921 | 947 | 938 | 99.98 |
| HEQLW1 | Harvest: Endosphere | High aridity-Live-Control | 52682 | 1612 | 1549 | 99.98 |
| HEQLW3 | Harvest: Endosphere | High aridity-Live-Control | 62609 | 1407 | 1317 | 99.99 |
| HEQLW5 | Harvest: Endosphere | High aridity-Live-Control | 71534 | 1620 | 1506 | 99.99 |
| HEQLW7 | Harvest: Endosphere | High aridity-Live-Control | 47328 | 1432 | 1382 | 99.97 |
| HEQLW9 | Harvest: Endosphere | High aridity-Live-Control | 37198 | 1202 | 1181 | 99.98 |
| HEQSD1 | Harvest: Endosphere | High aridity-Sterile-Water stress | 78413 | 730 | 678 | 100.00 |
| HEQSD3 | Harvest: Endosphere | High aridity-Sterile-Water stress | 44166 | 570 | 556 | 100.00 |
| HEQSD5 | Harvest: Endosphere | High aridity-Sterile-Water stress | 44845 | 660 | 643 | 100.00 |
| HEQSD7 | Harvest: Endosphere | High aridity-Sterile-Water stress | 39475 | 550 | 540 | 99.99 |
| HEQSD9 | Harvest: Endosphere | High aridity-Sterile-Water stress | 64530 | 744 | 708 | 100.00 |
| HEQSW1 | Harvest: Endosphere | High aridity-Sterile-Control | 78986 | 931 | 850 | 100.00 |
| HEQSW3 | Harvest: Endosphere | High aridity-Sterile-Control | 134585 | 1125 | 972 | 100.00 |
| HEQSW5 | Harvest: Endosphere | High aridity-Sterile-Control | 71079 | 880 | 821 | 99.99 |
| HEQSW7 | Harvest: Endosphere | High aridity-Sterile-Control | 88638 | 897 | 807 | 99.99 |
| HEQSW9 | Harvest: Endosphere | High aridity-Sterile-Control | 1207716 | 2004 | 1104 | 100.00 |
| HKLD1 | Harvest: Soil | Low aridity-Live-Water stress | 30853 | 1277 | 1259 | 99.95 |
| HKLD3 | Harvest: Soil | Low aridity-Live-Water stress | 24003 | 1042 | 1037 | 99.96 |
| HKLD5 | Harvest: Soil | Low aridity-Live-Water stress | 43611 | 1754 | 1697 | 99.96 |
| HKLD7 | Harvest: Soil | Low aridity-Live-Water stress | 41836 | 1630 | 1581 | 99.95 |
| HKLD9 | Harvest: Soil | Low aridity-Live-Water stress | 37829 | 1460 | 1418 | 99.97 |
| HKLDC1 | Harvest: Soil | Low aridity-Live-Water stress | 43877 | 1590 | 1538 | 99.96 |
| HKLDC2 | Harvest: Soil | Low aridity-Live-Water stress | 34773 | 1504 | 1484 | 99.98 |
| HKLDC3 | Harvest: Soil | Low aridity-Live-Water stress | 34695 | 1487 | 1466 | 99.97 |
| HKLW1 | Harvest: Soil | Low aridity-Live-Control | 28721 | 1354 | 1339 | 99.94 |
| HKLW3 | Harvest: Soil | Low aridity-Live-Control | 30237 | 1571 | 1550 | 99.94 |
| HKLW5 | Harvest: Soil | Low aridity-Live-Control | 24198 | 1199 | 1197 | 99.95 |
| HKLW7 | Harvest: Soil | Low aridity-Live-Control | 34795 | 1417 | 1392 | 99.98 |
| HKLW9 | Harvest: Soil | Low aridity-Live-Control | 23490 | 1238 | 1234 | 99.95 |
| HKLWC1 | Harvest: Soil | Low aridity-Live-Control | 35616 | 1708 | 1667 | 99.95 |
| HKLWC2 | Harvest: Soil | Low aridity-Live-Control | 48352 | 2027 | 1931 | 99.94 |
| HKLWC3 | Harvest: Soil | Low aridity-Live-Control | 61235 | 2187 | 2026 | 99.93 |
| HKSD2 | Harvest: Soil | Low aridity-Sterile-Water stress | 50003 | 506 | 494 | 100.00 |
| HKSD3 | Harvest: Soil | Low aridity-Sterile-Water stress | 52422 | 600 | 585 | 100.00 |
| HKSD5 | Harvest: Soil | Low aridity-Sterile-Water stress | 24395 | 1102 | 1100 | 99.98 |
| HKSD7 | Harvest: Soil | Low aridity-Sterile-Water stress | 48155 | 421 | 405 | 100.00 |
| HKSD9 | Harvest: Soil | Low aridity-Sterile-Water stress | 35447 | 478 | 469 | 100.00 |
| HKSDC1 | Harvest: Soil | Low aridity-Sterile-Water stress | 44247 | 499 | 493 | 100.00 |
| HKSDC2 | Harvest: Soil | Low aridity-Sterile-Water stress | 42123 | 423 | 415 | 100.00 |
| HKSW1 | Harvest: Soil | Low aridity-Sterile-Control | 45623 | 716 | 699 | 99.99 |
| HKSW3 | Harvest: Soil | Low aridity-Sterile-Control | 45095 | 794 | 771 | 99.99 |
| HKSW5 | Harvest: Soil | Low aridity-Sterile-Control | 28379 | 448 | 445 | 100.00 |
| HKSW7 | Harvest: Soil | Low aridity-Sterile-Control | 37602 | 638 | 623 | 99.99 |
| HKSW9 | Harvest: Soil | Low aridity-Sterile-Control | 41995 | 743 | 728 | 99.99 |
| HKSWC1 | Harvest: Soil | Low aridity-Sterile-Control | 31864 | 590 | 585 | 100.00 |
| HKSWC2 | Harvest: Soil | Low aridity-Sterile-Control | 84349 | 751 | 683 | 99.99 |
| HKSWC3 | Harvest: Soil | Low aridity-Sterile-Control | 35679 | 809 | 795 | 99.99 |
| HQLD1 | Harvest: Soil | High aridity-Live-Water stress | 39666 | 1640 | 1596 | 99.94 |
| HQLD3 | Harvest: Soil | High aridity-Live-Water stress | 37808 | 1550 | 1524 | 99.97 |
| HQLD5 | Harvest: Soil | High aridity-Live-Water stress | 34023 | 1393 | 1372 | 99.97 |
| HQLD7 | Harvest: Soil | High aridity-Live-Water stress | 25819 | 994 | 990 | 99.98 |
| HQLD9 | Harvest: Soil | High aridity-Live-Water stress | 45395 | 1775 | 1722 | 99.97 |
| HQLDC1 | Harvest: Soil | High aridity-Live-Water stress | 22929 | 902 | 898 | 99.98 |
| HQLDC2 | Harvest: Soil | High aridity-Live-Water stress | 36708 | 1178 | 1159 | 99.99 |
| HQLDC3 | Harvest: Soil | High aridity-Live-Water stress | 38789 | 505 | 499 | 100.00 |
| HQLW1 | Harvest: Soil | High aridity-Live-Control | 33967 | 1570 | 1543 | 99.96 |
| HQLW3 | Harvest: Soil | High aridity-Live-Control | 27578 | 1366 | 1351 | 99.95 |
| HQLW5 | Harvest: Soil | High aridity-Live-Control | 34749 | 1540 | 1512 | 99.98 |
| HQLW7 | Harvest: Soil | High aridity-Live-Control | 46137 | 1874 | 1806 | 99.96 |
| HQLW9 | Harvest: Soil | High aridity-Live-Control | 39626 | 1718 | 1671 | 99.95 |
| HQLWC1 | Harvest: Soil | High aridity-Live-Control | 23211 | 1126 | 1123 | 99.98 |
| HQLWC2 | Harvest: Soil | High aridity-Live-Control | 25180 | 1050 | 1044 | 99.97 |
| HQLWC3 | Harvest: Soil | High aridity-Live-Control | 30937 | 1569 | 1548 | 99.94 |
| HQSD1 | Harvest: Soil | High aridity-Sterile-Water stress | 54957 | 868 | 841 | 99.99 |
| HQSD3 | Harvest: Soil | High aridity-Sterile-Water stress | 34147 | 629 | 620 | 99.99 |
| HQSD5 | Harvest: Soil | High aridity-Sterile-Water stress | 31442 | 615 | 610 | 100.00 |
| HQSD7 | Harvest: Soil | High aridity-Sterile-Water stress | 41815 | 753 | 733 | 99.99 |
| HQSD9 | Harvest: Soil | High aridity-Sterile-Water stress | 31843 | 728 | 719 | 99.98 |
| HQSDC1 | Harvest: Soil | High aridity-Sterile-Water stress | 36623 | 716 | 706 | 99.99 |
| HQSDC2 | Harvest: Soil | High aridity-Sterile-Water stress | 48661 | 726 | 696 | 99.99 |
| HQSDC3 | Harvest: Soil | High aridity-Sterile-Water stress | 53185 | 827 | 787 | 99.99 |
| HQSW1 | Harvest: Soil | High aridity-Sterile-Control | 69545 | 1092 | 1016 | 99.99 |
| HQSW3 | Harvest: Soil | High aridity-Sterile-Control | 45073 | 843 | 827 | 100.00 |
| HQSW5 | Harvest: Soil | High aridity-Sterile-Control | 61584 | 1147 | 1067 | 99.99 |
| HQSW7 | Harvest: Soil | High aridity-Sterile-Control | 47507 | 947 | 909 | 99.99 |
| HQSW9 | Harvest: Soil | High aridity-Sterile-Control | 62946 | 1192 | 1126 | 99.98 |
| HQSWC1 | Harvest: Soil | High aridity-Sterile-Control | 36739 | 846 | 830 | 99.98 |
| HQSWC2 | Harvest: Soil | High aridity-Sterile-Control | 47051 | 895 | 864 | 99.99 |
| HQSWC3 | Harvest: Soil | High aridity-Sterile-Control | 26601 | 681 | 677 | 100.00 |
| HRKLD2 | Harvest: Rhizosphere | Low aridity-Live-Water stress | 90989 | 578 | 533 | 100.00 |
| HRKLD3 | Harvest: Rhizosphere | Low aridity-Live-Water stress | 27446 | 1396 | 1382 | 99.96 |
| HRKLD5 | Harvest: Rhizosphere | Low aridity-Live-Water stress | 69685 | 2618 | 2402 | 99.93 |
| HRKLD7 | Harvest: Rhizosphere | Low aridity-Live-Water stress | 32732 | 1636 | 1606 | 99.92 |
| HRKLD9 | Harvest: Rhizosphere | Low aridity-Live-Water stress | 39746 | 1812 | 1763 | 99.95 |
| HRKLW1 | Harvest: Rhizosphere | Low aridity-Live-Control | 29001 | 1475 | 1453 | 99.93 |
| HRKLW3 | Harvest: Rhizosphere | Low aridity-Live-Control | 22685 | 1178 | 1171 | 99.93 |
| HRKLW5 | Harvest: Rhizosphere | Low aridity-Live-Control | 315013 | 5197 | 3665 | 99.98 |
| HRKLW7 | Harvest: Rhizosphere | Low aridity-Live-Control | 18738 | 1096 | 1096 | 99.92 |
| HRKLW9 | Harvest: Rhizosphere | Low aridity-Live-Control | 43578 | 1792 | 1723 | 99.95 |
| HRKSD2 | Harvest: Rhizosphere | Low aridity-Sterile-Water stress | 30901 | 627 | 621 | 99.99 |
| HRKSD3 | Harvest: Rhizosphere | Low aridity-Sterile-Water stress | 55993 | 805 | 768 | 99.99 |
| HRKSD5 | Harvest: Rhizosphere | Low aridity-Sterile-Water stress | 39042 | 690 | 683 | 99.99 |
| HRKSD7 | Harvest: Rhizosphere | Low aridity-Sterile-Water stress | 84625 | 897 | 843 | 99.99 |
| HRKSD9 | Harvest: Rhizosphere | Low aridity-Sterile-Water stress | 43601 | 685 | 678 | 99.99 |
| HRKSW1 | Harvest: Rhizosphere | Low aridity-Sterile-Control | 50549 | 919 | 891 | 99.99 |
| HRKSW3 | Harvest: Rhizosphere | Low aridity-Sterile-Control | 62930 | 992 | 948 | 99.99 |
| HRKSW6 | Harvest: Rhizosphere | Low aridity-Sterile-Control | 42815 | 855 | 831 | 100.00 |
| HRKSW7 | Harvest: Rhizosphere | Low aridity-Sterile-Control | 27341 | 646 | 640 | 99.98 |
| HRKSW9 | Harvest: Rhizosphere | Low aridity-Sterile-Control | 71414 | 1098 | 1026 | 99.99 |
| HRQLD1 | Harvest: Rhizosphere | High aridity-Live-Water stress | 50524 | 1898 | 1821 | 99.95 |
| HRQLD3 | Harvest: Rhizosphere | High aridity-Live-Water stress | 53394 | 2081 | 1983 | 99.96 |
| HRQLD7 | Harvest: Rhizosphere | High aridity-Live-Water stress | 25897 | 1168 | 1156 | 99.93 |
| HRQLD9a | Harvest: Rhizosphere | High aridity-Live-Water stress | 30257 | 1460 | 1443 | 99.97 |
| HRQLD9b | Harvest: Rhizosphere | High aridity-Live-Water stress | 69980 | 2549 | 2358 | 99.94 |
| HRQLW1 | Harvest: Rhizosphere | High aridity-Live-Control | 24419 | 1175 | 1171 | 99.95 |
| HRQLW3 | Harvest: Rhizosphere | High aridity-Live-Control | 92232 | 2803 | 2494 | 99.96 |
| HRQLW5 | Harvest: Rhizosphere | High aridity-Live-Control | 50388 | 1993 | 1917 | 99.96 |
| HRQLW7 | Harvest: Rhizosphere | High aridity-Live-Control | 39387 | 1722 | 1679 | 99.94 |
| HRQSD1 | Harvest: Rhizosphere | High aridity-Sterile-Water stress | 29297 | 678 | 664 | 99.98 |
| HRQSD3 | Harvest: Rhizosphere | High aridity-Sterile-Water stress | 46910 | 889 | 861 | 99.99 |
| HRQSD5a | Harvest: Rhizosphere | High aridity-Sterile-Water stress | 31989 | 741 | 730 | 99.99 |
| HRQSD5b | Harvest: Rhizosphere | High aridity-Sterile-Water stress | 39546 | 1610 | 1567 | 99.94 |
| HRQSD7 | Harvest: Rhizosphere | High aridity-Sterile-Water stress | 31280 | 682 | 678 | 99.98 |
| HRQSD9 | Harvest: Rhizosphere | High aridity-Sterile-Water stress | 42059 | 843 | 826 | 99.99 |
| HRQSW1 | Harvest: Rhizosphere | High aridity-Sterile-Control | 32363 | 826 | 814 | 99.98 |
| HRQSW3 | Harvest: Rhizosphere | High aridity-Sterile-Control | 23358 | 596 | 596 | 99.99 |
| HRQSW5 | Harvest: Rhizosphere | High aridity-Sterile-Control | 33204 | 809 | 794 | 99.98 |
| HRQSW7 | Harvest: Rhizosphere | High aridity-Sterile-Control | 41720 | 828 | 800 | 99.99 |
| HRQSW9 | Harvest: Rhizosphere | High aridity-Sterile-Control | 32539 | 789 | 778 | 100.00 |
| RH.KLD1_t0 | Inital sampling: Soil | Low aridity-Live-Water stress | 60953 | 1623 | 1568 | 99.99 |
| RH.KLD3_t0 | Inital sampling: Soil | Low aridity-Live-Water stress | 22714 | 1033 | 1029 | 99.98 |
| RH.KLD5_t0 | Inital sampling: Soil | Low aridity-Live-Water stress | 35816 | 1177 | 1159 | 99.97 |
| RH.KLD7_t0 | Inital sampling: Soil | Low aridity-Live-Water stress | 72635 | 2010 | 1892 | 99.98 |
| RH.KLD9_t0 | Inital sampling: Soil | Low aridity-Live-Water stress | 87497 | 2502 | 2327 | 99.97 |
| RH.KLDC1_t0 | Inital sampling: Soil | Low aridity-Live-Water stress | 74099 | 2124 | 1985 | 99.98 |
| RH.KLDC2_t0 | Inital sampling: Soil | Low aridity-Live-Water stress | 131802 | 3122 | 2730 | 99.97 |
| RH.KLDC3_t0 | Inital sampling: Soil | Low aridity-Live-Water stress | 161802 | 3584 | 3008 | 99.98 |
| RH.KLW1_t0 | Inital sampling: Soil | Low aridity-Live-Control | 28941 | 958 | 954 | 99.98 |
| RH.KLW3_t0 | Inital sampling: Soil | Low aridity-Live-Control | 44740 | 1241 | 1213 | 99.98 |
| RH.KLW5_t0 | Inital sampling: Soil | Low aridity-Live-Control | 123453 | 2884 | 2541 | 99.98 |
| RH.KLW7_t0 | Inital sampling: Soil | Low aridity-Live-Control | 48284 | 1649 | 1611 | 99.98 |
| RH.KLW9_t0 | Inital sampling: Soil | Low aridity-Live-Control | 40050 | 1379 | 1355 | 99.98 |
| RH.KLWC1_t0 | Inital sampling: Soil | Low aridity-Live-Control | 75499 | 2190 | 2069 | 99.97 |
| RH.KLWC2_t0 | Inital sampling: Soil | Low aridity-Live-Control | 19381 | 804 | 804 | 99.98 |
| RH.KLWC3_t0 | Inital sampling: Soil | Low aridity-Live-Control | 26273 | 938 | 936 | 99.99 |
| RH.QLD1_t0 | Inital sampling: Soil | High aridity-Live-Water stress | 69492 | 1643 | 1577 | 99.99 |
| RH.QLD3_t0 | Inital sampling: Soil | High aridity-Live-Water stress | 63468 | 1683 | 1624 | 99.98 |
| RH.QLD5_t0 | Inital sampling: Soil | High aridity-Live-Water stress | 108670 | 2536 | 2297 | 99.98 |
| RH.QLD7_t0 | Inital sampling: Soil | High aridity-Live-Water stress | 120244 | 2554 | 2300 | 99.97 |
| RH.QLD9_t0 | Inital sampling: Soil | High aridity-Live-Water stress | 157847 | 2894 | 2533 | 99.99 |
| RH.QLDC1_t0 | Inital sampling: Soil | High aridity-Live-Water stress | 81346 | 2372 | 2189 | 99.97 |
| RH.QLDC2 | Inital sampling: Soil | High aridity-Live-Water stress | 88458 | 2452 | 2249 | 99.97 |
| RH.QLDC3_t0 | Inital sampling: Soil | High aridity-Live-Water stress | 79930 | 2271 | 2122 | 99.97 |
| RH.QLW1_t0 | Inital sampling: Soil | High aridity-Live-Control | 75329 | 1877 | 1791 | 99.98 |
| RH.QLW3_t0 | Inital sampling: Soil | High aridity-Live-Control | 65176 | 1658 | 1597 | 99.98 |
| RH.QLW5_t0 | Inital sampling: Soil | High aridity-Live-Control | 34610 | 1090 | 1084 | 99.99 |
| RH.QLW7_t0 | Inital sampling: Soil | High aridity-Live-Control | 37430 | 1115 | 1105 | 99.98 |
| RH.QLW9_t0 | Inital sampling: Soil | High aridity-Live-Control | 119396 | 2432 | 2210 | 99.98 |
| RH.QLWC1_t0 | Inital sampling: Soil | High aridity-Live-Control | 60499 | 1863 | 1804 | 99.98 |
| RH.QLWC2_t0 | Inital sampling: Soil | High aridity-Live-Control | 50116 | 1435 | 1383 | 99.98 |
| RH.QLWC3_t0 | Inital sampling: Soil | High aridity-Live-Control | 58752 | 1806 | 1737 | 99.99 |
| RH.QSD1_t0 | Inital sampling: Soil | High aridity-Sterile-Water stress | 190621 | 809 | 733 | 100.00 |
| RH.QSD5_t0 | Inital sampling: Soil | High aridity-Sterile-Water stress | 36924 | 708 | 708 | 100.00 |
| RH.QSDC2_t0 | Inital sampling: Soil | High aridity-Sterile-Water stress | 73073 | 180 | 177 | 100.00 |
| RH.QSDC3_t0 | Inital sampling: Soil | High aridity-Sterile-Water stress | 140619 | 963 | 920 | 100.00 |
| RH.QSW1_t0 | Inital sampling: Soil | High aridity-Sterile-Control | 165351 | 899 | 845 | 100.00 |
| RH.QSW3_t0 | Inital sampling: Soil | High aridity-Sterile-Control | 15571 | 71 | NA | 100.00 |
| RH.QSW5_t0 | Inital sampling: Soil | High aridity-Sterile-Control | 86846 | 509 | 494 | 100.00 |
| RH.QSW7_t0 | Inital sampling: Soil | High aridity-Sterile-Control | 26583 | 234 | 234 | 100.00 |
| RH.QSW9_t0 | Inital sampling: Soil | High aridity-Sterile-Control | 22405 | 232 | 232 | 100.00 |
| RH.QSWC1_t0 | Inital sampling: Soil | High aridity-Sterile-Control | 95538 | 413 | 402 | 100.00 |
| RH.QSWC2_t0 | Inital sampling: Soil | High aridity-Sterile-Control | 139229 | 428 | 406 | 100.00 |
| RH.QSWC3_t0 | Inital sampling: Soil | High aridity-Sterile-Control | 150424 | 563 | 536 | 100.00 |

**Table S5. Statistical output for randomised ANOVAs and subsequent pairwise comparisons.**

| **Diversity measure** | **Group 1** | **Group 2** | **P-value** | **Adjusted P-value (FDR)** |
| --- | --- | --- | --- | --- |
| Richness ~ Live experimental soils | Initial sampling: Soil | Harvest: Soil | 0.0582 | 0.1164 |
|  | Initial sampling: Soil | Harvest: Rhizosphere | 0.8573 | 0.8573 |
|  | Initial sampling: Soil | Harvest: Endosphere | 0.0329 | 0.1164 |
|  | Harvest: Soil | Harvest: Rhizosphere | 0.0817 | 0.12255 |
|  | Harvest: Soil | Harvest: Endosphere | 0.4250 | 0.5100 |
|  | Harvest: Rhizosphere | Harvest: Endosphere | 0.0421 | 0.1164 |
| Richness ~ Sterilised experimental soils | Initial sampling: Soil | Harvest: Soil | 0.0708 | 0.1656 |
|  | Initial sampling: Soil | Harvest: Rhizosphere | 0.0103 | 0.0618 |
|  | Initial sampling: Soil | Harvest: Endosphere | 0.1128 | 0.1692 |
|  | Harvest: Soil | Harvest: Rhizosphere | 0.2881 | 0.34572 |
|  | Harvest: Soil | Harvest: Endosphere | 0.5598 | 0.5598 |
|  | Harvest: Rhizosphere | Harvest: Endosphere | 0.0828 | 0.1656 |
| Richness ~ Live control soils | Initial sampling: Soil | Harvest: Soil | 0.0229 | - |
| Richness ~ Sterilised control soils | Initial sampling: Soil | Harvest: Soil | NS ANOVA | - |
| Effective no. ASVs ~ Live experimental soils | Initial sampling: Soil | Harvest: Soil | 0.0257 | 0.03855 |
|  | Initial sampling: Soil | Harvest: Rhizosphere | 0.7778 | 0.7778 |
|  | Initial sampling: Soil | Harvest: Endosphere | 0.0000 | 0.0000 |
|  | Harvest: Soil | Harvest: Rhizosphere | 0.1270 | 0.1524 |
|  | Harvest: Soil | Harvest: Endosphere | 0.0000 | 0.0000 |
|  | Harvest: Rhizosphere | Harvest: Endosphere | 0.0000 | 0.0000 |
| Effective no. ASVs ~ Sterilised experimental soils | Initial sampling: Soil | Harvest: Soil | 0.5865 | 0.7038 |
|  | Initial sampling: Soil | Harvest: Rhizosphere | 0.7855 | 0.7855 |
|  | Initial sampling: Soil | Harvest: Endosphere | 0.0033 | 0.0088 |
|  | Harvest: Soil | Harvest: Rhizosphere | 0.2182 | 0.3273 |
|  | Harvest: Soil | Harvest: Endosphere | 0.0044 | 0.0088 |
|  | Harvest: Rhizosphere | Harvest: Endosphere | 0.0000 | 0.0000 |
| Effective no. ASVs ~ Live control soils | Initial sampling: Soil | Harvest: Soil | 0.0086 | - |
| Effective no. ASVs ~ Sterilised control soils | Initial sampling: Soil | Harvest: Soil | NS ANOVA | - |

**Table S6. Statistical output for randomised linear mixed effects models, and randomised ANOVAs.** The effect of each treatment variables on *T. triandra* associated with bacterial alpha diversity under live and sterile communities. ‘*’ denotes p values <0.05, ‘**’ denotes p values <0.01, ‘***’ denotes p values <0.001. †Only output related to aridity variables is reported.

| **Formula** | **Random effects** | **Permutations** | **Fixed effect** | **T-statistic** | | **P-value** |
| --- | --- | --- | --- | --- | --- | --- |
| Effective No. ASVs ~ Aridity (Live only) | Compartment + Water stress | 10,000 | Aridity | | -0.156 | 0.875 |
| Effective No. ASVs ~ Water stress (Live only) | Aridity + Compartment | 10,000 | Water stress | | -1.049 | 0.312 |
| Effective No. ASVs ~ Aridity (Sterilised only) | Compartment + Water stress | 10,000 | Aridity | | -1.774 | 0.086 |
| Effective No. ASVs ~ Water stress (Sterilised only) | Aridity + Compartment | 10,000 | Water stress | | 0.211 | 0.833 |
| Effective No. ASVs ~ sterilisation (Initial sampling only) | Water stress + Aridity | 10,000 | Sterilisation | | -8.760 | <0.001*** |
| Effective No. ASVs ~ sterilisation (Harvest only) | Compartment + Water stress + Aridity | 10,000 | Sterilisation | | -17.296 | <0.001*** |
| Effective No. ASVs ~ Plant presence (live only) | Compartment + Water stress + Aridity | 10,000 | Plant presence | | -0.567 | 0.584 |
| Effective No. ASVs ~ Plant presence (sterilised only) | Compartment + Water stress + Aridity | 10,000 | Plant presence | | 1.159 | 0.255 |
| Richness ~ Aridity (Live only) | Compartment + Water stress | 10,000 | Aridity | | -0.442 | 0.6717 |
| Richness ~ Water stress (Live only) | Aridity + Compartment | 10,000 | Water stress | | -0.948 | 0.3584 |
| Richness ~ Aridity (Sterilised only) | Compartment + Water stress | 10,000 | Aridity | | -1.774 | 0.0947 |
| Richness ~ Water stress (Sterilised only) | Aridity + Compartment | 10,000 | Water stress | | -1.988 | 0.0546 |
| Richness ~ sterilisation (Initial sampling only) | Water stress + Aridity | 10,000 | Sterilisation | | -7.294 | <0.001*** |
| Richness ~ sterilisation (Harvest only) | Compartment + Water stress + Aridity | 10,000 | Sterilisation | | -12.68 | <0.001*** |
| Richness ~ Plant presence (live only) | Compartment + Water stress + Aridity | 10,000 | Plant presence | | -0.644 | 0.5156 |
| Richness ~ Plant presence (sterilised only) | Compartment + Water stress + Aridity | 10,000 | Plant presence | | 1.171 | 0.2567 |

**Table S7. Differential abundance output showing log fold change in bacterial phyla across each plant timepoint and compartment (soil, rhizosphere and endosphere), and each treatment (microbial aridity, sterilisation and water stress).** All comparisons are made to high aridity, live, control watering treatments. Reference comparisons represent LFC change from grand mean. ‘NS’ indicate non-significant LFC results. ‘*’ represents p<0.05, ‘**’ represents p<0.01, and ‘***’ represents p<0.001.

| Compartment | Bacterial phylum | Reference (High aridity: Live: Control) | ~Low aridity | ~Sterile | ~Water-stress |
| --- | --- | --- | --- | --- | --- |
| Initial sampling: Soil~ | Actinobacteriota | 0.523* | -0.668** | -1.332** | 0.208 NS |
|  | Firmicutes | -0.185 NS | -1.209** | 2.161** | 0.214 NS |
|  | Verrucomicrobiota | 0.553* | -1.021** | -0.941 NS | 0.205 NS |
|  | Bacteroidota | -0.676* | 0.739 NS | 0.839 NS | 0.467 NS |
|  | Gemmatimonadota | 0.535 NS | -0.668* | -1.499 NS | 0.279 NS |
|  | Myxococcota | -0.145 NS | 0.887** | -0.58 NS | -0.084 NS |
|  | Cyanobacteria | 0.441 NS | -1.171* | -1.364* | 0.51 NS |
|  | Armatimonadota | 0.336 NS | -1.549** | -0.076 NS | 0.485 NS |
|  | RCP2-54 | -1.775*** | 2.26*** | 2.408** | 0.032 NS |
|  | Nitrospirota | 0.294 NS | -1.554** | 0.017 NS | 0.327 NS |
|  | WPS-2 | 1.35** | -2.489*** | -0.735 NS | -0.439 NS |
|  | Methylomirabilota | -2.001*** | 2.544*** | 0.895 NS | 0.444 NS |
|  | Dependentiae | -1.175** | 0.832 NS | 2.427** | 1.14 NS |
|  | Deinococcota | -0.553 NS | -0.377 NS | 2.325* | -0.19 NS |
|  | WS2 | -0.706 NS | -0.371 NS | 3.278** | 0.831 NS |
|  | Entotheonellaeota | 0.45 NS | -1.108* | 0.084 NS | -0.088 NS |
| Harvest: Soil~ | Actinobacteriota1 | 0.028 NS | -0.047 NS | -0.757*** | 0.773*** |
|  | Fibrobacterota | -0.421* | 0.788*** | 0.958*** | -0.662*** |
|  | Firmicutes1 | -1.14*** | -0.263 NS | 2.853*** | 0.124 NS |
|  | Verrucomicrobiota1 | -0.255 NS | -0.037 NS | 0.754* | 0.02 NS |
|  | Acidobacteriota | 0.714*** | -0.058 NS | -0.9*** | -0.524** |
|  | Bacteroidota1 | -0.692* | 0.721* | 1.476*** | -0.425 NS |
|  | Myxococcota1 | -0.101 NS | 0.506* | 0.157 NS | -0.273 NS |
|  | Sumerlaeota | 0.436 NS | -1.308*** | 2.008*** | -1.101*** |
|  | Desulfobacterota | -0.251 NS | 0.664* | 1.363*** | -1.192*** |
|  | Dadabacteria | -0.811*** | -0.312 NS | 2.132*** | -2.077*** |
|  | Armatimonadota1 | 0.54 NS | -0.721* | 0.367 NS | -0.657* |
|  | Patescibacteria | 0.75* | 0.129 NS | -0.524 NS | -1.028** |
|  | RCP2-541 | 0.858** | -0.469 NS | -0.428 NS | -0.736* |
|  | Bdellovibrionota | -0.385 NS | 0.078 NS | 1.64*** | -0.353 NS |
|  | Hydrogenedentes | -0.1 NS | 0.443 NS | 1.071*** | -1.533*** |
|  | Nitrospirota1 | 0.515* | -0.534* | -0.146 NS | -0.414 NS |
|  | WPS-21 | 0.683* | -0.466 NS | 0.195 NS | -1.076*** |
|  | Methylomirabilota1 | -0.811*** | 1.979*** | -1.154*** | -0.4* |
|  | Latescibacterota | -1.434*** | 2.576*** | 0.047 NS | -0.38 NS |
|  | Dependentiae1 | 0.404 NS | -0.62* | 1.081*** | -0.858** |
|  | Deinococcota1 | -0.721*** | 0.205 NS | 1.592*** | -0.27 NS |
|  | MBNT15 | 0.37 NS | -0.057 NS | 0.696** | -1.152*** |
|  | WS21 | 0.933*** | -0.85*** | 0.025 NS | -0.931*** |
|  | NB1-j | 0.518** | 0.614** | -1.226*** | -1.09*** |
|  | Entotheonellaeota1 | 0.318 NS | -0.526* | -0.093 NS | 0.003 NS |
|  | Planctomycetota | -0.188 NS | 0.336 NS | 0.669* | -0.352 NS |
|  | SAR324_clade(Marine_group_B) | -0.748*** | 0.648** | 1.09*** | -1.18*** |
|  | Elusimicrobiota | 0.514* | -0.549* | 0.017 NS | -0.535** |
| Harvest: Rhizosphere~ | Actinobacteriota2 | -0.398 NS | 0.06 NS | -0.024 NS | 0.916*** |
|  | Fibrobacterota1 | -1.378*** | 1.057** | 0.95** | 0.726 NS |
|  | Firmicutes2 | -1.217** | 0.079 NS | 1.741*** | 0.562 NS |
|  | Chloroflexi | 0.559** | -0.15 NS | -0.543** | -0.187 NS |
|  | Acidobacteriota1 | 0.616*** | 0.476* | -1.217*** | -0.218 NS |
|  | Bacteroidota2 | -0.225 NS | 0.166 NS | 0.486* | -0.024 NS |
|  | Gemmatimonadota1 | 0.344 NS | -0.826*** | -0.041 NS | 0.364 NS |
|  | Myxococcota2 | -0.737 NS | 0.806* | -0.54 NS | 1.367*** |
|  | Sumerlaeota1 | 0.994** | -0.952* | 0.703 NS | -1.499*** |
|  | Desulfobacterota1 | -0.471 NS | 0.712** | 0.576* | -0.165 NS |
|  | Dadabacteria1 | -3.156*** | 0.943*** | 3.805*** | -1.41*** |
|  | Armatimonadota2 | 0.914*** | -0.959*** | -0.253 NS | -0.384 NS |
|  | Patescibacteria1 | 0.71 NS | -0.237 NS | -1.821*** | 0.885 NS |
|  | RCP2-542 | 1.277*** | -0.562 NS | -1.352*** | -0.355 NS |
|  | Hydrogenedentes1 | 0.045 NS | 1.075** | 0.509 NS | -1.645*** |
|  | Methylomirabilota2 | -0.348 NS | 1.109*** | -1.815*** | 0.244 NS |
|  | Latescibacterota1 | -0.819** | 3.518*** | -0.682* | -0.554 NS |
|  | Dependentiae2 | 0.396 NS | -0.141 NS | 0.565* | -1.004*** |
|  | WS22 | 0.805** | -0.361 NS | -1.867*** | 0.131 NS |
|  | NB1-j1 | 1.459*** | 0.052 NS | -2.722*** | -0.612* |
|  | Spirochaetota | 1.105*** | -1.185** | -0.861** | -1.042** |
|  | SAR324_clade(Marine_group_B)1 | -0.685* | 0.639* | 0.861** | -0.447 NS |
| Harvest: Endosphere~ | Fibrobacterota2 | -1.827*** | 0.272 NS | 3.456*** | 0.06 NS |
|  | Firmicutes3 | -1.726*** | 0.071 NS | 3.49*** | 0.032 NS |
|  | Proteobacteria | -0.36* | 0.327 NS | 0.833*** | -0.298 NS |
|  | Chloroflexi1 | 0.455 NS | 0.223 NS | -0.415 NS | -0.577* |
|  | Verrucomicrobiota2 | -0.553 NS | 0.336 NS | 1.768*** | -0.856* |
|  | Bacteroidota3 | 0.083 NS | -0.322 NS | 0.795** | -0.498 NS |
|  | Gemmatimonadota2 | 0.493 NS | -0.969* | -0.544 NS | 0.668 NS |
|  | Myxococcota3 | -0.287 NS | 0.452 NS | 0.591* | -0.328 NS |
|  | Sumerlaeota2 | 0.747* | -1.847*** | 0.674* | -0.73 NS |
|  | Desulfobacterota2 | -1.484*** | 1.074*** | 2.049*** | -0.24 NS |
|  | Cyanobacteria1 | -0.68* | 0.128 NS | 1.706*** | -0.333 NS |
|  | Armatimonadota3 | 0.585* | -1.053** | 0.323 NS | -0.332 NS |
|  | Patescibacteria2 | 1.289*** | -0.663 NS | -1.966*** | -0.107 NS |
|  | Bdellovibrionota1 | -0.913*** | 0.736* | 1.883*** | -0.651 NS |
|  | Hydrogenedentes2 | -0.581 NS | -0.707* | 1.493*** | -0.179 NS |
|  | Nitrospirota2 | -0.213 NS | 0.124 NS | -0.682** | 0.239 NS |
|  | WPS-22 | -0.406 NS | -0.176 NS | 1.323*** | -0.561 NS |
|  | Dependentiae3 | -0.099 NS | -0.3 NS | 1.169*** | -0.602 NS |
|  | Deinococcota2 | -1.232*** | 1.959*** | -0.726* | -0.131 NS |
|  | MBNT151 | -1.384*** | 0.652* | 1.303*** | -0.272 NS |
|  | WS23 | -0.49* | 0.53* | 0.909*** | -0.071 NS |
|  | NB1-j2 | -1.739*** | 2.091*** | -1.114*** | -0.031 NS |
|  | Spirochaetota1 | -0.333 NS | 0.545* | 0.795** | -2.101*** |
|  | Planctomycetota1 | -0.801** | 0.636* | 1.288*** | -0.562 NS |
|  | SAR324_clade(Marine_group_B)2 | 0.049 NS | -0.501* | -0.103 NS | 0.47 NS |

**Table S8. Differential abundance output showing log fold change in bacterial phyla across each control pot (plant-absent) across and each treatment (microbial aridity, sterilisation and water stress).** All comparisons are made to high aridity, live, control watering treatments. Reference comparisons represent LFC change from grand mean. ‘NS’ indicate non-significant LFC results, ‘*’ represents p<0.05, ‘**’ represents p<0.01, and ‘***’ represents p<0.001.

| Compartment | Bacterial phylum | Reference (High aridity: Live: Control) | ~Low aridity | ~Sterile | ~Water-stress |
| --- | --- | --- | --- | --- | --- |
| Initial sampling: Soil~ | Actinobacteriota | 0.015 NS | -0.182 NS | -0.662* | 0.318 NS |
|  | Firmicutes | -0.508 NS | -0.925 NS | 1.95* | 0.356 NS |
|  | Chloroflexi | -0.568** | 0.466 NS | 0.739* | 0.195 NS |
|  | Bacteroidota | -0.872** | 0.567 NS | 1.372* | 0.37 NS |
|  | Gemmatimonadota | 0.13 NS | -0.597 NS | -0.581* | 0.334 NS |
|  | Myxococcota | -0.15 NS | 1.262* | -1.13 NS | -0.122 NS |
|  | Cyanobacteria | -0.667 NS | -0.834 NS | 1.306* | 1.199* |
|  | RCP2-54 | -2.273** | 2.828* | -0.268 NS | 0.854 NS |
|  | WPS-2 | 0.886* | -1.927** | -1.235 NS | -0.17 NS |
|  | Methylomirabilota | -1.548** | 3.001** | 0.722 NS | 0.138 NS |
|  | Entotheonellaeota | -0.642 NS | 0.274 NS | -0.435 NS | 1.197* |
|  | Planctomycetota | -1.294** | 0.902 NS | 1.385 NS | 1.002 NS |
| Harvest: Soil~ | Actinobacteriota1 | -0.247 NS | 0.09 NS | -0.437 NS | 1.03** |
|  | Fibrobacterota | -0.752* | 0.877 NS | 1.233** | -1.013** |
|  | Firmicutes1 | -2.417** | -0.014 NS | 3.701*** | 1.085 NS |
|  | Proteobacteria | -0.099 NS | 0.13 NS | 0.657* | -0.414 NS |
|  | Acidobacteriota | 0.633 NS | 0.416 NS | -0.476 NS | -1.097* |
|  | Bacteroidota1 | -1.128* | 1.375** | 2.027*** | -0.878* |
|  | Sumerlaeota | 0.064 NS | -0.847 NS | 2.013** | -1.142* |
|  | Desulfobacterota | -0.493 NS | 0.819 NS | 1.484* | -1.481* |
|  | Dadabacteria | 1.258** | -0.005 NS | -0.021 NS | -2.353*** |
|  | Armatimonadota | 0.369 NS | 0.151 NS | 0.589 NS | -1.382** |
|  | Patescibacteria | 0.914 NS | 1.123 NS | -1.147* | -1.72** |
|  | Bdellovibrionota | -0.676* | 0.376 NS | 1.381*** | -0.176 NS |
|  | Hydrogenedentes | -0.177 NS | 1.28 NS | 0.37 NS | -2.497*** |
|  | Nitrospirota | 0.926* | -0.147 NS | -0.592 NS | -1.198** |
|  | Latescibacterota | -2.879*** | 5.28*** | 0.161 NS | -1.641** |
|  | Dependentiae | 0.161 NS | 0.051 NS | 1.026** | -1.261** |
|  | MBNT15 | 0.571 NS | 0.207 NS | 0.516 NS | -2.14*** |
|  | WS2 | 0.203 NS | 0.346 NS | 0.258 NS | -1.262** |
|  | NB1-j | 1.802** | -0.209 NS | -2.81*** | -1.864** |
|  | Planctomycetota1 | 0.069 NS | 0.565 NS | 0.632 NS | -1.184* |
|  | SAR324_clade  (Marine_group_B) | -0.634 NS | 0.811 NS | 0.559 NS | -2.007** |

**References**

Searle, R., Malone, B., Wilford, J., Austin, J., Ware, C., Webb, M., Roman Dobarco, M., & Van Niel, T. (2022) TERN Digital Soil Mapping Raster Covariate Stacks. CSIRO.
